# Exploring the Biochemical Mechanism Beyond the Cytotoxic Activity of Sesquiterpene Lactones from Sicilian Accession of *Laserpitium siler* Subsp. *siculum* (Spreng.) Thell

**DOI:** 10.3390/plants14213289

**Published:** 2025-10-28

**Authors:** Alessandro Vaglica, Antonella Maggio, Chiara Occhipinti, Natale Badalamenti, Marianna Lauricella, Maurizio Bruno, Antonella D’Anneo

**Affiliations:** 1 Department of Biological, Chemical and Pharmaceutical Sciences and Technologies (STEBICEF), University of Palermo, Viale delle Scienze, 90128 Palermo, Italy; alessandro.vaglica@unipa.it (A.V.); chiara.occhipinti@unipa.it (C.O.); natale.badalamenti@unipa.it (N.B.); maurizio.bruno@unipa.it (M.B.); antonella.danneo@unipa.it (A.D.); 2 Department of Biomedicine, Neurosciences and Advanced Diagnostics (BIND), Section of Biochemistry, University of Palermo, Via del Vespro 129, 90127 Palermo, Italy; marianna.lauricella@unipa.it

**Keywords:** Apiaceae, *Laserpitium siler* subsp. *siculum*, sesquiterpene lactones, guaianolides, breast cancer, MDA-MB-231 cells

## Abstract

*Laserpitium siler* subsp. *siculum* (Apiaceae) is a Mediterranean plant with a long history of traditional medicinal use. In this study, the chemical composition and anticancer potential of three novel (and one new to the genus) sesquiterpene lactones (SLs) isolated from its roots were investigated. The structural characterization, carried out through NMR and HPLC-MS analyses, identified unique guaianolide-type lactones. The biological activity of these compounds was evaluated in vitro using MDA-MB-231 cells, a triple-negative breast cancer (TNBC) cell line. Cell viability assays demonstrated that all SLs tested reduced TNBC cell viability in a dose- and time-dependent manner, with SL-1 exhibiting the highest cytotoxicity. Light microscopy analyses and acridine orange/ethidium bromide staining confirmed the induction of apoptotic cell death, further supported by Western blot analyses showing caspase-3 activation and PARP-1 cleavage. Additional experiments indicated that SL-1 induced oxidative stress, as evidenced by increased ROS production and upregulation of the levels of the antioxidant enzymes MnSOD and HO-1. Moreover, JC-1 staining and Western blot analyses revealed mitochondrial membrane depolarization as well as a significant reduction in VDAC-1 expression, suggesting mitochondrial dysfunction as a key event in the cytotoxic mechanism. These findings highlight *L*. *siler* subsp. *siculum* as a promising source of bioactive compounds with anticancer potential. The ability of its sesquiterpene lactones to induce oxidative stress and mitochondrial impairment provides new insights into their mode of action, supporting further research into their therapeutic applications for TNBC treatment.

## 1. Introduction

The genus *Laserpitium* L. (Apiaceae) consists of approximately twenty aromatic perennial herbaceous species, predominantly endemic to the Mediterranean region and Southwest Asia, although their distribution extends from the Canary Islands to Iran and Siberia [1]. The genus is particularly diverse in mountainous areas of southern and central Europe, where around fourteen species are listed in *Flora Europaea* [2]. *Laserpitium siler* L., also known as laserwort or *Siler montanum* Crantz, is one of the most well-known species. It is found in rocky meadows at altitudes of approximately 1400 m in the submeridional-montane regions of central Europe.

The plants of genus *Laserpitium* are characterized by spindle-shaped roots that transition into a short, thickened rhizome. They produce small white or pink flowers and schizocarpic fruits rich in essential oils [3]. These plants have long been used in traditional European medicine, with *Laserpitium* species providing remedies for a wide range of health issues. For instance, the herb of *Laserpitium carduchorum* Hedge & Lamond has been used as a spice and for treating urinary tract infections, while gum resins from the roots of *Laserpitium gallicum* L. are used in managing tumors and warts [4,5].

Roots and rhizomes of various *Laserpitium* species, such as *Laserpitium latifolium* L., known as “Radix Gentianae albae” [6], and *Laserpitium siler*, traditionally called “raskovnik” [7], have been used for their digestive, diuretic, and anti-inflammatory properties. These plants are also employed for treating gastrointestinal disorders, heart and liver dysfunctions, tuberculosis, rheumatism, and as topical treatments for pruritic dermatomycoses [8,9]. In the Balkans, fresh roots of *L. siler* have been used as an antidote for snake bites and to treat cataracts [10], while in the Alps, they were used for preparing tonics that refreshed and strengthened the body and also in mouthwashes for treating toothache [11].

The extensive use of *Laserpitium* species in traditional herbal medicine has spurred scientific investigations into their chemical composition and bioactivity. Recent studies have highlighted the cytotoxic, anti-inflammatory, antibacterial, and antifungal activities of *Laserpitium* species [11,12]. For example, extracts from *Laserpitium zernyi* Hayek and *Laserpitium ochridanum* Micevski have demonstrated cytotoxic effects on human breast cancer cell lines [8].

*Laserpitium* species are also known to produce essential oils rich in monoterpenes, with *α*/*β*-pinene, sabinene, limonene, and chamazulene being major components [13,14]. Among these, *L. siler* exhibits various chemotypes, with subsp. *siculum* characterized by perillaldehyde/chamazulene/sabinene and subsp. *montanum* by *trans*-anethole, limonene, and sabinene [15]. *L. ochridanum*, endemic to North Macedonia, contains *α*-pinene, chamazulene, and *α*-bisabolol in its roots and rhizomes [8]. In addition to essential oils, these plants are rich in flavonoids, phenylpropanoids, sesquiterpenoids, coumarins, and daucans, which exhibit a variety of bioactivities such as antioxidant, antimicrobial, and anti-inflammatory effects [16,17].

Among the bioactive compounds found in *Laserpitium* species, sesquiterpene lactones, particularly guaianolides, are of particular interest due to their significant pharmacological activities. These compounds are key to the chemotaxonomy of the genus, as they are rich in phenolic compounds and sesquiterpene lactones, notably slovanolides, which are found in *L. siler*, *L. zernyi*, *L. marginatum*, and *L. ochridanum* [18]. These lactones have demonstrated anticancer, anti-inflammatory, and antimicrobial properties [19]. *L. siler* and *L. zernyi* accumulate guaianolide lactones that are structurally distinct from those found in the Asteraceae family, thus serving as phytochemical markers for distinguishing the relationships within the Apiaceae family [20].

While much attention has been given to the bioactive properties of sesquiterpene lactones, including their anti-inflammatory, immunomodulatory, and antimicrobial effects [16,20,21], their potential as anticancer agents is still an emerging area of research. A recent review highlighted the diverse biological activities of sesquiterpene lactones, with particular emphasis on their anticancer potential [22].

Breast cancer represents the most diagnosed neoplasia in women and the main cancer-related death. Among the different breast cancer subtypes, triple-negative breast cancer (TNBC) has been identified as the most aggressive type of breast cancer with poor prognosis [23].

Although many efforts have been made to identify selective treatment options for patients with advanced TNBC, this form of tumor still represents a therapeutically challenging subtype of breast cancer, largely due to its high metastatic potential, frequent recurrence, and resistance to conventional chemotherapies [24]. Current research emphasizes the importance of disrupting mitochondrial metabolism and ROS-mediated pathways to overcome therapy resistance and attenuate TNBC cell survival under hypoxic conditions [25]. Notably, previous studies demonstrated that Parthenolide, a sesquiterpene lactone extracted from the medical herb feverfew *Tanacetum parthenium* and its soluble derivative DMAPT (Dimethylamino Parthenolide), exerted a marked cytotoxic effect on MDA-MB-231 cells, a triple-negative breast cancer cell line. This effect was primarily correlated with generation of ROS, which in turn was responsible for induction of autophagy, dissipation of mitochondrial membrane potential (Δψm) and a caspase-independent form of death [26]. Additionally, recent studies have shown that guaianolide-type sesquiterpene lactones such as Ambrosin selectively induce apoptosis in drug-resistant TNBC cells through the mitochondrial pathway, involving increased ROS production, caspase cascade activation, and suppression of anti-apoptotic proteins like Bcl-2. This was accompanied by a significant loss in mitochondrial membrane potential and inhibition of Akt/*β*-catenin signaling, highlighting the relevance of targeting redox-sensitive and mitochondrial pathways in TNBC therapy [27].

Given the wide chemical diversity of *Laserpitium* species and the promising bioactivities of their sesquiterpene lactones, research into their anticancer properties, particularly in the context of TNBC, is of great interest. In light of these observations, with the aim to identify specific and selective natural compounds targeting TNBC, our study aimed at investigating the anti-tumor potential of *Laserpitium siler* subsp. *siculum* sesquiterpene lactones and explore their mode of action.

Since no data is available on the chemical characterization and biological properties of the Sicilian variety of *Laserpitium siler* subsp. *siculum* to date, we undertook this research. Our study focused on the chemical composition of the plant as well as the analysis of its potential anticancer properties, making it a relevant target for future investigation. Indeed, guaianolide lactones isolated from these subspecies present unique structural features that could offer novel mechanisms of action in cancer therapy.

## 2. Results and Discussion

### 2.1. Metabolomic Analysis

The roots of *L*. *siler* subsp. *siculum* were harvested in May 2020, during the flowering stage, and extracted in accordance with the methodology outlined in Section 3, resulting in the isolation of four sesquiterpene lactones (SL1–SL4) and one triterpenoid (**5**).

**Figure 1:**
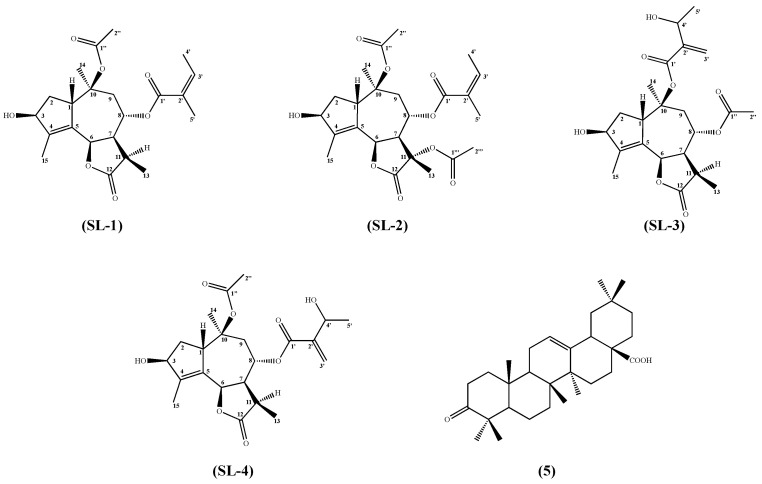
Structures of compounds.

**SL-1** is an amorphous white solid with molecular formula C_22_H_30_O_7_, deducible by positive HRESI-MS peak at *m/z* 429.1909 [M + Na]^+^ (theoretical mass 429.1889 Da). The ^1^H-NMR and ^13^C-NMR spectra, respectively, exhibited the distinctive signals characteristic of a lactone type carbonyl (*δ_C_* = 177.98, C-12). This in conjunction with the double bond between C-4 and C-5, as evidenced by the signals at *δ_C_* = 148.21 and 131.64; and with the methyl group C-13 in *β* at *δ_C_* = 11.28, led to the conclusion that the sesquiterpene lactone skeleton was present. All of the aforementioned confirmations were achieved through HMBC correlation between C-4 and H-2, between C-5 and H-7 (in order to ascertain the position of the double bond), and between C-13 and H-7 (for the methyl group). Building on these HMBC correlations, and the ^1^H-^1^H COSY coupling (H-1 with H-2, H-2 with H-3, H-6 with H-7, H-7 with H-8, etc.) the remaining data further confirmed the sesquiterpene framework (see Supplementary Materials). The substituents identified as a hydroxyl group in C-3 (*δ_C_* = 79.31, *δ_H_* = 4.77), an acetate group in C-10, and an angeloyl group in C-8. This was achieved through a comparison of the signals with those reported in the literature and with HMBC correlations between H-8 and C-1′. To further validate the proposed structure and stereochemistry, key NOESY correlations were observed between the methyl protons at H-14 and H-3, between H-14 and H-6, and between H-14 and H-7. Additionally, the large vicinal coupling constant between H-7 and H-8 (*J* = 10.5 Hz), together with the NOESY contacts involving H-8, unequivocally supports the configuration of the stereocenters depicted in Figure 1. A 3D model showing these principal correlations is provided in the Supplementary Materials. SL-1 (Figure 1), known as (1*S*,3*R*,6*R*,7*S*,8*S*,10*S*,11*R*)-3-hydroxy-10-acetoxy-8-angeloyloxyguaia-4(15),5(6)-dien-12,6-olide, has not been previously isolated from plants belonging to the *Laserpitium* genera. The structural elucidation of SL-1 and its guaianolide-type sesquiterpene lactone skeleton was supported by comparison with spectral data of known sesquiterpenes and by the sole report of this compound from the aerial parts of *Seseli vayredanum* Font Quer [28].

SL-2 was obtained as a white powder and assigned to the molecular formula C_24_H_32_O_9_ by HRESI-MS analysis (*m/z* 487.1939 [M + Na]^+^; theoretical mass 487.1941 Da). As with the initial compound, most signals observed for compound SL-2 were comparable to those previously documented. However, the presence of a new acetate substituent, identified by the variation in the C-11 signal (*δc* = 79.19), necessitated a separate analysis. The two acetate groups were located mainly by NOESY experiments. The first acetate at C-10 was identified by NOESY cross-peaks between H-1 and the acetate methyl protons H-2″, and by the absence of any NOESY contacts between H-1 and the protons of the C-8 substituent. Given the geometry of the guaianolide framework, this is the only spatial contact expected for this position, and it is indeed observed. Instead, H-8 itself showed NOESY correlations with both H-1 and H-2″ confirming the previously assigned configuration of C-8. After establishing the acetate at C-10, NOESY correlations were examined to identify the α-oriented substituent at C-8, applying the same stereochemical considerations used for SL-1. Correlations between H-9*β* and the acetate methyls H-2″, as well as between H-9*β* and the angeloyl methyl protons H-4′, assigned the angeloyl group to C-8. Consequently, the second acetate was placed at C-11, since its methyl protons H-2‴ correlates with H-6 and H-7, and did not interact with H-1, whereas H-13 displayed a clear NOESY cross-peak with H-1 and H-8. (See 3D model in Supplementary Materials). Indeed, the stereochemistry of C-11 was determined through the observation of a NOESY correlation between H-13, H-8 and H-1. The angeloyl moiety was once again confirmed to be in the *α* position through the analysis of the coupling constants. SL-2 (Figure 1), ((1*S*,3*R*,6*R*,7*S*,8*S*,10*S*,11*S*)-3-hydroxy-10,11-diacetoxy-8-angeloyloxyguaia-4(15),5(6)-dien-12,6-olide), has not been previously isolated from other plants.

SL-3 and SL-4 were obtained as a white powder in a mixture (ratio 1:1) that could not be separated using either column chromatography, preparative TLC or other RP-HPLC/chiral methods. Nevertheless, it was possible to characterize and identify them through HPLC-MS and the different integrations of the peaks in NMR spectroscopy. The mixture was consistently observed in repeated chromatographic runs and after storage of the sample solution (24–48 h at room temperature), with no change in the SL-3/SL-4 ratio, thus excluding degradation or isomerization artifacts. For both, the molecular formula C_22_H_30_O_8_ was assigned by HRESI-MS analysis (*m/z* 445.1833 [M + Na]^+^; theoretical mass 445.1837 Da). The two products exhibited a sesquiterpene lactone structure identical to that observed in the two preceding products, except for the two substituents in C-10 and C-8, which were previously occupied by an acetate and angeloyl groups and now by an acetate and a 3-hydroxy-2-methylenebutanoate substituent group. This distinction was identified through the appearance of new signals (*δc-*_1__′_ = 164.96, *δc-*_2′_ = 143.97, *δc-*_3′_ = 123.81, *δc-*_4′_ = 67.04, *δc-*_5′_ = 22.62). The two compounds differ from each other because of the two substituents, which appear to be inverted in their respective structures. It was possible to discern the two structures thanks to the HMBC correlations of the two different carbonyls of substituting groups with the H-8. The stereochemistry was confirmed in a manner analogous to that employed for the preceding compounds. The isolation of compounds SL-3 ((1*S*,3*R*,6*R*,7*S*,8*S*,10*S*,11*R*)-3-hydroxy-8-acetoxy-10-[2-(1-hydroxyethyl)acryloyloxy]guaia-4(15),5(6)-dien-12,6-olide) and SL-4 ((1*S*,3*R*,6*R*,7*S*,8*S*,10*S*,11*R*)-3-hydroxy-10-acetoxy-8-[2-(1-hydroxyethyl)acryloyloxy]guaia-4(15),5(6)-dien-12,6-olide) represents a novel occurrence.

Compound 5 was assigned the molecular formula C_30_H_46_O_3_, supported by HPLC-MS analysis (*m*/*z* 455.3521 [M + H]^+^, theoretical mass 455.3524 Da). The compound was identified as the known triterpenoid oleanonic acid by comparison with literature data [29] and on the basis of complete NMR characterization: ^13^C-NMR (30 signals) and ^1^H-NMR, with diagnostic resonances including H-12 (*δ* = 5.28), C-28 (*δ* = 182.99), and C-3 (*δ* = 217.61), and HMBC correlations consistent with the proposed structure. Full NMR table and spectra are provided in the Supplementary Materials.

### 2.2. Bioassays

**Figure 2:**
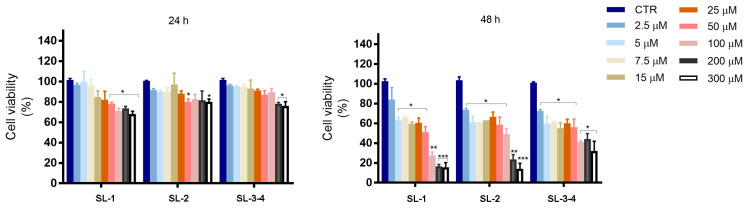
The treatment with *L. siler* SLs reduced MDA-MB-231 cell viability. Time and dose-dependent effects of SLs ) were analyzed by MTT assay after 24 h and 48 h of treatment, respectively. Cells were seeded in 96 well plates (8 × 10^3^) and incubated for the indicated times with different doses of compounds. Data are reported as mean value ± SD. (*) *p* < 0.05, (**) *p* < 0.01, (***) *p* < 0.001 respect to the control.

To assess the biological effects of *Laserpitium siler* sesquiterpene lactones (SL-1, SL-2, SL-3 and SL-4), triple negative breast cancer cells (MDA-MB-231) were treated with increasing concentrations (2.5–300 μM) of compounds for 24 and 48 h, respectively. As shown in Figure 2, all SLs reduced cell viability in a dose- and time-dependent manner, with modest effects at 24 h even with the highest doses used (100, 200 and 300 μM), reaching a percentage of viable cells of about 80%. Prolonging the treatment up to 48 h, all three sesquiterpene lactones showed moderate cytotoxic effects at the lower doses tested (2.5–25 μM), while significant differences were observed at higher concentrations. **SL-1** was the most effective, reducing viability by 50% at 50 μM and 72.5% at 100 μM, while SL-2 and SL-3–4 showed weaker effects.

Indeed, the comparison of the half maximal inhibitory concentration (IC_50_) for all three SLs (Table 1) after 48 h of treatment showed that SL-1 is the most efficacious sesquiterpene lactone with a lower IC_50_ (50 ± 5 μM) than SL-2 (IC_50_ = 90 ± 3 μM) and SL-3–4 (IC_50_ = 250 ± 2 μM).

Light microscopy analyses confirmed these findings, showing a decrease in cell number as early as 24 h after treatment with SL-1 to SL-3–4 (Figure S6). However, the four sesquiterpene lactones displayed distinct effects on cell morphology. Indeed, SL-1-induced cytotoxic effect observed at 24 h with a 100 μM dose was related to the induction of cell death (Figure S6A), whereas the action of SL-2 and SL-3–4 was primarily associated with a decrease in cell number, suggesting an inhibition of cell proliferation rather than direct cytotoxicity. Prolonging the treatment of cells up to 48 h, the analysis clearly indicated SL-1 as the most effective among the sesquiterpene lactones tested.

**Figure 3:**
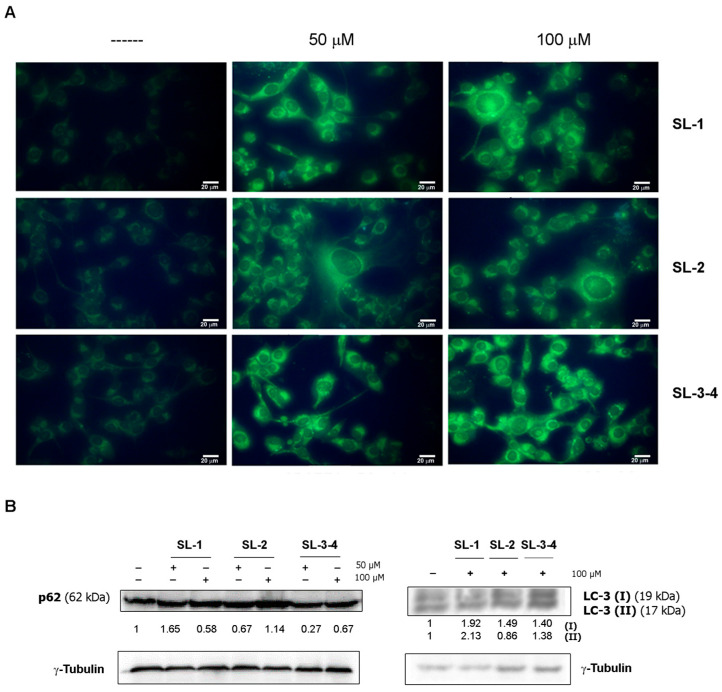
*Laserpitium siler* sesquiterpene lactone treatment induced autophagy at 24 h. (**A**) Cells were treated for 24 h in the presence of SL-1, SL-2 and SL-3–4, then autophagy was detected by monodansylcadaverine (MDC) staining as reported in the Materials and Methods section. All images were acquired with exposure times of 200 msec at 400× original magnification using an inverted fluorescent OPTIKA IM3FL4 microscope equipped with OPTIKA PROVIEW imaging system (OPTIKA Srl, Italia), scale bar 20 μm. (**B**) Autophagy markers (p62 and LC3) were evaluated by Western blotting analysis. After 24 h of incubation with sesquiterpene lactones, cells were lysed and analyzed for the proteins of interest using specific antibodies. Densitometry analysis was performed to identify the relative expression of proteins of interest, normalized to *γ*-Tubulin for loading control. Data reported below each lane are representative of three independent experiments with similar results and show protein abundance relative to untreated control (n = 3).

The cytotoxic effect of *Laserpitium siler* SLs was linked to the induction of both autophagic cell death and apoptosis. To further explore the underlying mechanisms, additional studies were conducted to determine whether the analyzed sesquiterpene lactones could trigger autophagy. Cells were treated with compounds for 24 h followed by monodansylcadaverine (MDC) staining, a green fluorescent dye able to bind to lipids present on the membrane of autophagosomes. As shown in Figure 3, all sesquiterpene lactones induced the appearance of “dot-like” structures, typical of autophagic vacuoles, in cells treated for 24 h.

**Figure 4:**
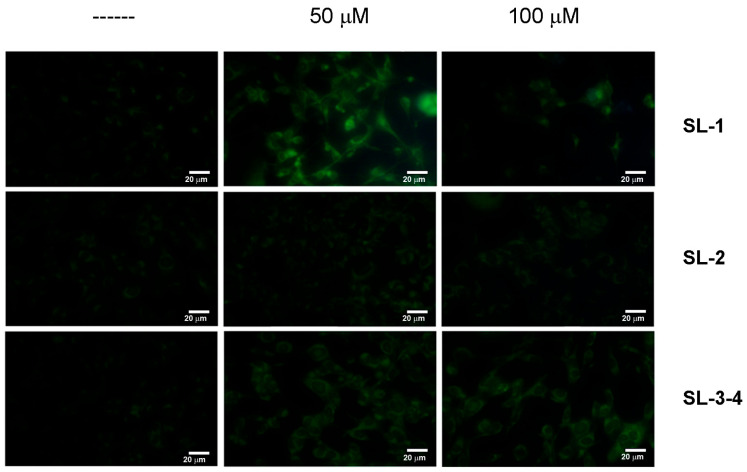
Autophagic vacuoles disappeared at longer times of incubation with sesquiterpene lactones. Cells were treated for 48 h in the presence of SL-1, SL-2 and SL-3–4, then the presence of autophagic vacuoles was evaluated by fluorescence microscopy using MDC staining. All images were captured with exposure times of 200 msec at 400× original magnification by a fluorescent microscope (OPTIKA IM3FL4) equipped with an imaging system (OPTIKA PROVIEW, OPTIKA Srl, Italia), scale bar 20 μm.

Such an aspect was also confirmed by Western blotting analyses, showing the increase in p62 and LC3 II levels, two known autophagic markers [30], in the cells incubated in the presence of sesquiterpene lactones. However, this effect was only observed after 24 h of treatment. Prolonging the treatment up to 48 h, the disappearance of autophagic vacuoles (Figure 4) occurred and noticeable changes in cellular morphology were observed, suggesting a shift toward an alternative cell death pathway.

To further investigate the observed changes, acridine orange (AO)/ethidium bromide (EB) double staining was performed in MDA-MB-231 cells treated for 48 h with SLs. These fluorochromes selectively stain live and dead cells, allowing fluorescence microscopy to differentiate between early apoptotic cells (yellow-green AO nuclear staining) and late apoptotic cells (orange nuclear EB staining).

**Figure 5:**
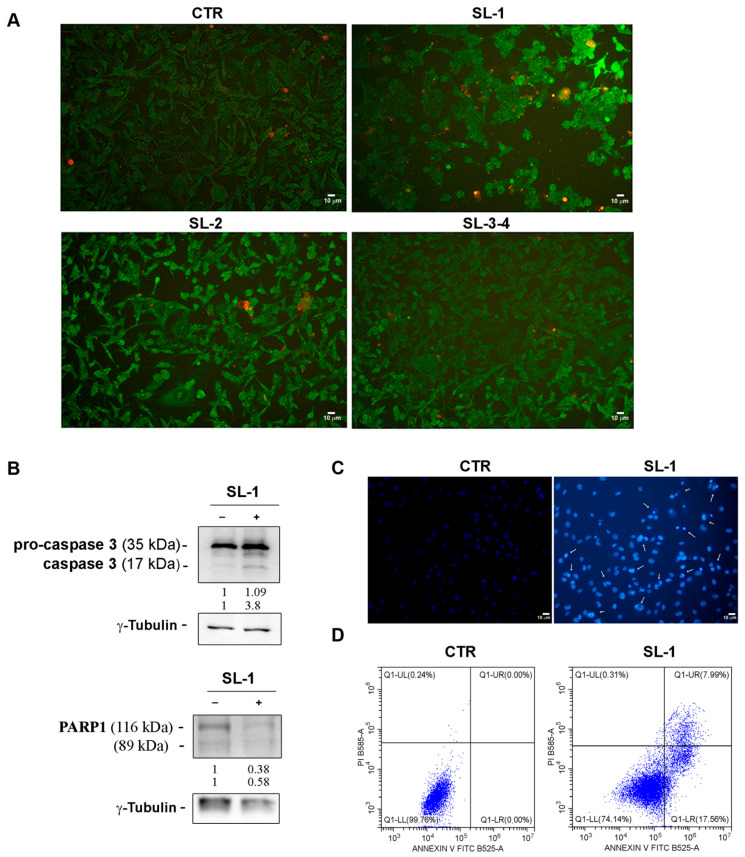
SL-1 treatment induced apoptotic cell death. (**A**) Apoptotic cell death analysis was performed by acridine orange/ethidium bromide staining. Cells were treated for 48 h with sesquiterpene lactones as reported in the Materials and Methods. Images were captured with exposure times of 200 msec at 100 original magnification by inverted fluorescence microscope OPTIKA IM3FL4 endowed with OPTIKA PROVIEW imaging system (OPTIKA Srl, Italia) (scale bar 10 µm). (**B**) Western blotting analyses of apoptotic markers (caspase-3 and PARP-1) analyzed in compound SL-1-treated cells for 48 h. Densitometry analysis was performed to identify the relative expression of proteins of interest, normalized to *γ*-Tubulin for loading control. Data reported below each lane are representative of three independent experiments with similar results and report protein abundance relative to untreated control (n = 3). (**C**) Analysis of MDA-MB-231 cells after Hoechst 33342 vital staining. Pictures were taken at 200× original magnification (exposure times of 200 msec) using inverted fluorescence microscope OPTIKA IM3FL4 endowed with OPTIKA PROVIEW imaging system (OPTIKA Srl, Italia), scale bar 10 µm. Arrows indicate condensed chromatin. (**D**) Flow cytometry analysis by Annexin V-FICT/PI double staining of apoptotic cells induced by compound SL-1 treatment. Data was acquired by CytoFLEX instrument (Beckman Coulter, Brea, CA, USA) using CytExpert software v2.5.

Fluorescence microscopy images obtained by the merging of red and green fluorescence, as shown in Figure 5, revealed that the presence of reddish-orange spots in SL-1 treated cells, suggesting the induction of apoptotic cell demise. Differently, a very modest effect was observed when the cells were incubated in the presence of the other sesquiterpene lactones SL-2 and SL-3–4 (Figure 5A).

Therefore, the activity of SL-1 was further investigated by performing Western blotting analyses on caspase-3 and PARP-1 (Figure 5B), two known markers of apoptosis. As reported in Figure 5B, SL-1 treatment caused the fragmentation of procaspase-3 (35 kDa) in the active form (17 kDa) promoting the execution phase of apoptosis, and the concomitant reduction in levels of PARP-1, a typical substrate of active caspase-3 [31]. These molecular changes provided evidence that the treatment with **SL-1** induces death by apoptosis at 48 h.

Such observations were also confirmed by Hoechst staining (Figure 5C) demonstrating that SL-1 treatment caused DNA condensation, a typical apoptotic event. Furthermore, flow cytometry investigations, conducted using Annexin V/PI double-staining assay to quantitatively analyze the extent of apoptotic cell death in SL-1-treated cells (Figure 5D) revealed that almost 26% of treated cells resulted positive to apoptosis, amounting to 17.56% in early-stage apoptosis (Annexin V^+^/PI^−^) and 7.99% in late-stage apoptosis (Annexin V^+^/PI^+^).

To further clarify the mode of action of SL-1, its ability to induce ROS production and disrupt mitochondrial membrane potential was investigated. Reactive oxygen species (ROS) can trigger autophagy and, under prolonged stress, may lead to apoptosis if oxidative damage exceeds the cell’s repair capacity [32]. To assess ROS generation, cells were incubated with SL-1 for 1, 2, and 4 h and the analysis was performed by using H_2_DCFDA, a ROS-sensitive dye.

**Figure 6:**
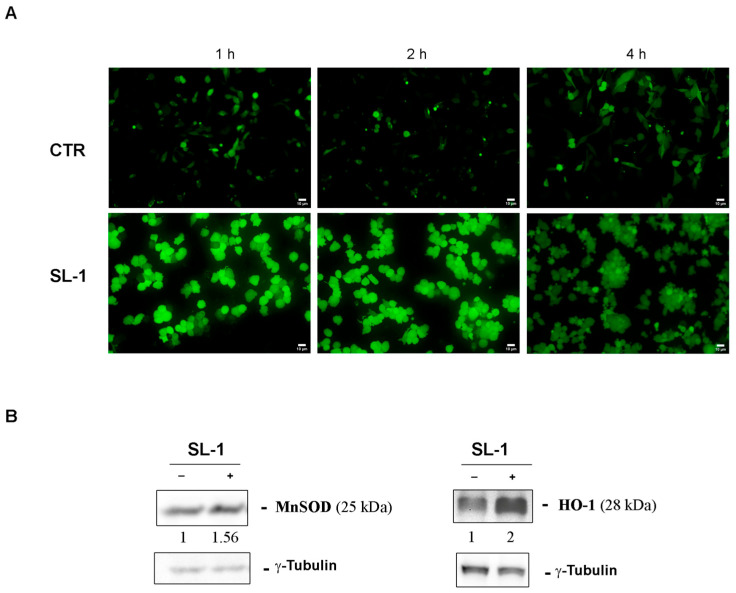
SL-1 effect was mediated by oxidative stress. (**A**) ROS analysis was performed by H_2_DCFDA fluoro-probe staining. Cells were treated with **SL-1** for the indicated times and the assay was performed as reported in the Materials and Methods. Images were captured with exposure times of 200 msec at 200× original magnification by inverted fluorescence microscope OPTIKA IM3FL4 endowed with OPTIKA PROVIEW imaging system (OPTIKA Srl, Italia) (scale bar 10 µm). (**B**) Western blotting analyses of oxidative stress associated enzymes (MnSOD and HO-1) analyzed in SL-1-treated cells. Densitometry analysis was performed to identify the relative expression of proteins of interest, normalized to *γ*-Tubulin for loading control. Data reported below each lane are representative of three independent experiments with similar results and report protein abundance relative to untreated control (n = 3).

As shown in Figure 6A, treated cells exhibited intense green fluorescence compared to untreated controls, indicating ROS production as early as 1–2 h after incubation with SL-1.

These findings were further supported by Western blot analyses of the antioxidant enzymes superoxide dismutase (MnSOD) and heme oxygenase 1 (HO-1) (Figure 6B). SL-1 treatment led to a significant increase in the expression of both enzymes at 24 h, suggesting the activation of cellular defense mechanisms involved in the antioxidant response.

Since excessive ROS accumulation can lead to mitochondrial membrane potential dissipation, resulting in mitochondrial dysfunction and apoptosis [33,34,35], mitochondrial integrity was assessed using JC-1 staining. The results revealed a significant loss of mitochondrial membrane potential in SL-1-treated cells at 24 h, indicating mitochondrial dysfunction as a key event precursor to apoptosis [36].

**Figure 7:**
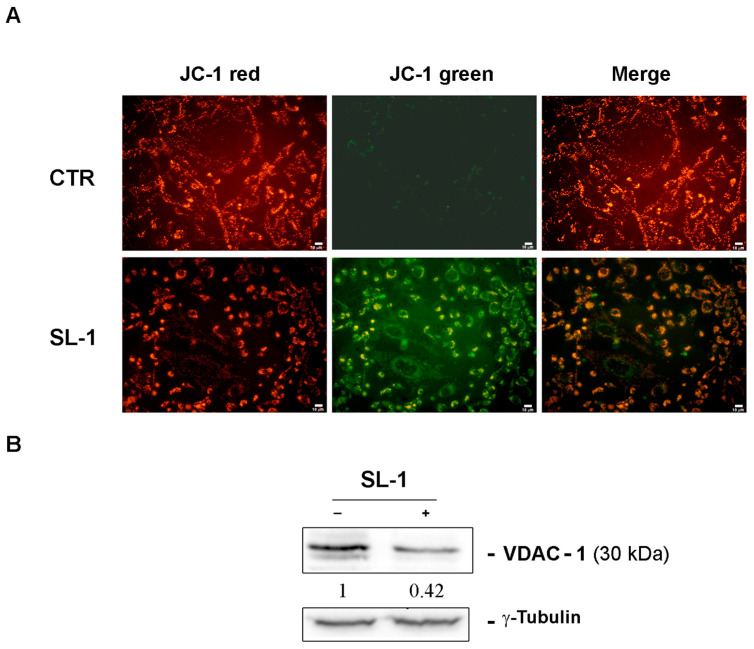
SL-1 caused mitochondrial membrane impairment. SL-1 caused mitochondrial membrane impairment (**A**). The analysis of mitochondrial membrane dissipation was evaluated by JC-1 staining. The image shows the loss of red fluorescence (JC-1 aggregates refer to a high mitochondrial membrane potential) and the acquisition of green fluorescence (JC1 monomers refer to a low mitochondrial membrane potential) in MDA-MB-231 cells exposed to SL-1 treatment. For these analyses cells were treated with SL-1 and pictures (scale bar 10 µm) were captured with exposure times of 200 msec at 200× original magnification by inverted fluorescence microscope OPTIKA IM3FL4 endowed with OPTIKA PROVIEW imaging system (OPTIKA Srl, Italia). (**B**) Western blotting analyses of VDAC-1 were performed in SL-1-treated cells. Densitometry analysis was performed to identify the relative expression of proteins of interest, normalized to *γ*-Tubulin for loading control. Data reported below each lane are representative of three independent experiments with similar results and show protein abundance relative to untreated control (n = 3).

To further support these findings, Western blot analyses showed a reduction in VDAC-1 expression following **SL-1** treatment (Figure 7). As a key mitochondrial membrane protein regulating metabolite and ion exchange between the cytosol and mitochondria [37], the decreased VDAC-1 levels suggest its involvement in mitochondrial destabilization, contributing to apoptosis and impaired energy homeostasis.

When compared with well-known guaianolide sesquiterpene lactones such as parthenolide and ambrosin, SL-1 exhibited a comparable cytotoxic potency against MDA-MB-231 cells, showing an IC_50_ value of 50 ± 5 µM after 48 h of treatment, consistent with the low micromolar range reported for these reference compounds in TNBC models [26,27]. Similarly to parthenolide and ambrosin, the cytotoxic effect of SL-1 appears to be mediated by excessive ROS generation, loss of mitochondrial membrane potential, and activation of apoptotic pathways, suggesting the involvement of a common redox-sensitive mitochondrial mechanism. However, the distinctive structural features of SL-1, including subtle variations in the guaianolide core and, in particular, the presence of an angeloyl substituent at C-8 and an acetoxy group at C-10 (Figure 1), may contribute to its enhanced activity profile. These substituents can modulate the compound’s lipophilicity and electronic distribution, potentially enhancing its ability to penetrate cellular membranes and interact with mitochondrial targets. Therefore, the observed cytotoxic potency of SL-1 likely arises from a combination of these structural modifications, which may fine-tune its redox behavior and apoptotic potential, supporting a structure–activity relationship consistent with that of other bioactive guaianolides.

## 3. Materials and Methods

### 3.1. Plant Material

The specimen of *L*. *siler* subsp. *siculum* was collected in the Madonie Mountains, Sicily, Italy, on dolomitic slopes in the vicinity of Contrada Quacella (37°50′44.94″ N; 14°01′12.73″ E), at an elevation of 1500 m above sea level, in May 2020. A voucher specimen of the population under analysis was deposited in the herbarium of the University of Palermo, Italy (Voucher no. 109716).

### 3.2. Extraction and Isolation

Following the removal of the leaves and stems by manual means, the roots of *L*. *siler* subsp. *siculum* (1400 g) were meticulously washed, cut into thin strips and frozen at a temperature of −20 °C. Subsequently, the vegetable material was subjected to a freeze-drying cycle for a period of one week, with the objective of eliminating all the water present. This process resulted in the production of 800 g of dry vegetable material. The freeze-dried product was finely ground through a ceramic mortar and extracted with acetone (2 L × 3 times) at room temperature and in the dark. Following filtration, the solvent was evaporated at reduced pressure and at temperatures below 40 °C to prevent the heat from causing changes to any thermolabile substances present. The extract (7 g) was subjected to column chromatography (5 × 60 cm) using silica gel deactivated with 15% cold water as the stationary phase, with mixtures of cyclohexane and ethyl acetate in the ratio 8:2, 7:3, 6:4, 2:8, and 1:9 (*v*/*v*) used as the eluent. At the conclusion of this process, a few fractions (principally from 6:4 ratio) were isolated, some of which exhibited the potential for interesting products as observed through thin-layer chromatography (TLC). The general anisaldehyde/H_2_SO_4_ system was employed as the detection system.

### 3.3. Structural Identification

Merck No. 7734 silica gel (70–230 mesh ASTM) deactivated with 15% water was used for column chromatography. A CoolSafe 4–15 L Freeze Dryers tool (LaboGene A/S, Allerød, Denmark) with a capacity of 4 L was used for freezing drying. The characterization of the metabolites was carried out by ^1^H-NMR and ^13^C-NMR spectroscopy with a Brucker Avance II spectrometer (Bruker Corporation, Billerica, MA, USA) with a rotating MAS probe at 15 KHz operating at 400 MHz for recording the ^1^H-NMR spectra, and at 400 MHz for the ^13^C spectra. DEPT, ^1^H-^1^H-COSY, HMBC, HSQC, DEPT and NOESY experiments were performed using Bruker microprograms (Bruker Corporation, Billerica, MA, USA). The samples to be analyzed were brought into solution with CDCl_3_. For all spectra, the coupling constants *J* are expressed in Hz. The different products were subjected to Agilent 1260 Infinity HPLC–MS analysis. A 2.1 mm × 50 mm Zorbax Extend C_18_ reversed phase column with 1.8 μm particle size was used, with a 4 mm × 3 mm Phenomenex C_18_ safety column. The flow rate was 0.5 mL/min and the column temperature was set to 25 °C. The eluents formic acid/water as phase A, and formic acid/methanol as phase B. The injection volume was 3 μL. Everything was monitored via MS TIC. Mass spectra were obtained with the Agilent 6540 uHD (Agilent Technologies, Santa Clara, CA, USA)accurate mass Q-TOF spectrometer equipped with an AJS ESI dual working resource operating in positive mode and in negative mode. N_2_ was used as a desolvating gas at 320 °C with a flow rate of 10 L/min. The nebulizer was set at 35 psig. The jacket gas temperature was set at 350 °C with a flow rate of 11 L/min. A potential of 3.5 kV on the capillary was used for the positive ion mode. The fragmenter was selected at 75 V. The mass spectra were recorded in the 100–1000 *m/z* range. Polarimetric measurements were performed for the determination of stereochemistry. A Jasco P1010 digital polarimeter (Jasco, Tokyo, Japan) was used for this analysis.

Five products were isolated, two pure sesquiterpene lactones: SL-1 and SL-2, a mix of two isomers SL-3 and SL-4, and a triterpenoid 5 (purities > 95% via HPLC).

SL-1: (300 mg) amorphous solid, [*α*]^25^_D_ = −5.80 (c 0.05, CHCl_3_); ^1^H NMR (CDCl_3_, 400 MHz): *δ* 6.05 (1H, qq, J = 7.2, 1.3 Hz, H-3′), 5.34 (1H, d, J = 6.3 Hz, H-6), 5.23 (1H, dd, J = 9.6, 9.6 Hz, H-8), 4.77 (1H, dd, J = 6.8, 6.4 Hz, H-3), 3.27 (1H, br d, J = 8.3 Hz, H-1), 2.99 (1H, dq, J = 7.6 Hz, H-11), 2.80 (1H, ddd, J = 9.6, 7.6, 6.3 Hz, H-7), 2.66 (1H, d, J = 15.4 Hz, H-9β), 2.59 (1H, dd, J = 13.8, 6.4 Hz, H-2β), 2.09 (3H, s, H-2″), 1.96 (3H, dq, J = 7.2, 1.3 Hz, H-4′), 1.86 (3H, m, H-15), 1.84 (3H, dq, J = 1.3, 1.3 Hz, H-5′), 1.77 (1H, dd, J = 15.4, 9.6 Hz, H-9α), 1.70 (1H, ddd, J = 13.8, 8.3, 6.8 Hz, H-2α), 1.39 (3H, s, H-14), 1.22 (3H, d, J = 7.6 Hz, H-13); ^13^C NMR (CDCl_3_, 400 MHz): *δ* 177.98 (C-12), 171.40 (C-2″), 166.14 (C-1′), 148.21 (C-4), 138.90 (C-3′), 131.64 (C-5), 127.37 (C-2′), 83.97 (C-10), 79.31 (C-3), 75.12 (C-6), 66.28 (C-8), 49.98 (C-1), 48.47 (C-7), 43.82 (C-9), 39.92 (C-11), 37.95 (C-2), 22.61 (C-2″), 21.64 (C-14), 20.31 (C-5′), 15.64 (C-4′), 11.28 (C-13), 11.21 (C-15).

SL-2: (100 mg) amorphous solid, [*α*]^25^_D_ = −7.90 (c 0.05, CHCl_3_); ^1^H NMR (CDCl_3_, 400 MHz): *δ* 6.12 (1H, qq, J = 7.2, 1.3 Hz, H-3′), 5.64 (1H, d, J = 10.5 Hz, H-6), 5.50 (1H, dd, J = 10.5, 10.5 Hz, H-8), 4.74 (1H, dd, J = 7.1, 6.4 Hz, H-3), 3.54 (1H, dd, J = 10.5, 10.5 Hz, H-7), 3.18 (1H, br d, J = 8.3 Hz, H-1), 2.90 (1H, d, J = 15.6 Hz, H-9β), 2.52 (1H, dd, J = 14.1, 6.4 Hz, H-2β), 2.12 (3H, s, H-2‴), 2.08 (3H, s, H-2″), 1.98 (3H, dq, J = 7.2, 1.3 Hz, H-4′), 1.93 (3H, br s, H-15), 1.85 (3H, dq, J = 1.3, 1.3 Hz, H-5′), 1.73 (1H, ddd, J = 14.1, 8.3, 7.1 Hz, H-2α), 1.50 (1H, dd, J = 15.6, 10.5 Hz, H-9α), 1.57 (3H, s, H-13), 1.31 (3H, s, H-14); ^13^C NMR (CDCl_3_, 400 MHz): *δ* 178.98 (C-1″), 173.96 (C-12), 170.10 (C-1‴), 166.23 (C-1′), 150.61 (C-4), 139.44 (C-3′), 130.90 (C-5), 127.13 (C-2′), 83.50 (C-10), 79.69 (C-3), 79.19 (C-11), 73.15 (C-6), 66.19 (C-8), 52.46 (C-1), 48.99 (C-7), 40.62 (C-9), 36.46 (C-2), 22.56 (C-2‴), 20.83 (C-2″), 20.75 (C-13), 20.29 (C-5′), 20.20 (C-14), 15.71 (C-4′), 12.16 (C-15).

*SL-3*: (150 mg with SL-4) amorphous solid; ^1^H NMR (CDCl_3_, 400 MHz): *δ* 6.10 (1H, d, J = 1.1 Hz, H-3′a), 5.85 (1H, d, J = 1.1 Hz, H-3′b), 5.35 (1H, d, J = 6.3 Hz, H-6), 5.25 (1H, dd, J = 9.6, 9.6 Hz, H-8), 4.78 (1H, dd, J = 6.8, 6.4 Hz, H-3), 4.60 (1H, brq, J = 6.5 Hz, H-4′), 3.24 (1H, br d, J = 8.3 Hz, H-1), 3.00 (1H, dq, J = 7.6 Hz, H-11), 2.84 (1H, ddd, J = 9.6, 7.6, 6.3 Hz, H-7), 2.68 (1H, d, J = 15.6 Hz, H-9β), 2.62 (1H, dd, J = 13.8, 6.4 Hz, H-2β), 2.08 (3H, s, H-2″), 1.87 (3H, m, H-15), 1.73 (1H, dd, J = 15.6, 9.6 Hz, H-9α), 1.70 (1H, ddd, J = 13.8, 8.3, 6.8 Hz, H-2α), 1.39 (3H, s, H-14), 1.36 (3H, d, J = 6.5 Hz, H-5′), 1.20 (3H, d, J = 7.6 Hz, H-13); ^13^C NMR (CDCl_3_, 400 MHz): *δ* 177.78 (C-12), 170.97 (C-1″), 164.96 (C-1′), 148.54 (C-4), 143.97 (C-2′), 131.38 (C-5), 123.81 (C-3′), 83.81 (C-10), 79.22 (C-3), 74.94 (C-6), 67.36 (C-8), 67.04 (C-4′), 49.82 (C-1), 48.51 (C-7), 43.81 (C-9), 39.92 (C-11), 37.97 (C-2), 22.62 (C-5′), 22.20 (C-2″), 21.63 (C-14), 11.22 (C-13), 11.22 (C-15).

SL-4: amorphous solid; ^1^H NMR (CDCl_3_, 400 MHz): *δ* 6.11 (1H, d, J = 1.1 Hz, H-3′a), 5.85 (1H, d, J = 1.1 Hz, H-3′b), 5.35 (1H, d, J = 6.3 Hz, H-6), 5.26 (1H, dd, J = 9.6, 9.6 Hz, H-8), 4.78 (1H, dd, J = 6.8, 6.4 Hz, H-3), 4.60 (1H, brq, J = 6.5 Hz, H-4′), 3.24 (1H, br d, J = 8.3 Hz, H-1), 3.00 (1H, dq, J = 7.6 Hz, H-11), 2.84 (1H, ddd, J = 9.6, 7.6, 6.3 Hz, H-7), 2.66 (1H, d, J = 15.6 Hz, H-9β), 2.62 (1H, dd, J = 13.8, 6.4 Hz, H-2β), 2.08 (3H, s, H-2″), 1.87 (3H, m, H-15), 1.75 (1H, dd, J = 15.6, 9.6 Hz, H-9α), 1.70 (1H, ddd, J = 13.8, 8.3, 6.8 Hz, H-2α), 1.39 (3H, s, H-14), 1.37 (3H, d, J = 6.5 Hz, H-5′), 1.21 (3H, d, J = 7.6 Hz, H-13); ^13^C NMR (CDCl_3_, 400 MHz): *δ* 177.78 (C-12), 170.90 (C-1″), 165.02 (C-1′), 148.54 (C-4), 143.97 (C-2′), 131.38 (C-5), 123.93 (C-3′), 83.81 (C-10), 79.22 (C-3), 74.94 (C-6), 67.21 (C-8), 67.04 (C-4′), 49.82 (C-1), 48.51 (C-7), 43.71 (C-9), 39.92 (C-11), 37.97 (C-2), 22.58 (C-5′), 22.20 (C-2″), 21.63 (C-14), 11.22 (C-13), 11.22 (C-15).

5: (300 mg) amorphous solid, [*α*]^25^_D_ = −7.90 (c 0.05, CHCl_3_); ^1^H NMR (CDCl_3_, 400 MHz): *δ* 5.29 (1H, t, H-12), 2.85 (1H, dd, J = 11.4 Hz, H-18), 2.54–2.37 (2H, m, H-2), 1.99 (1H, m, H-16b), 1.99 (1H, m, H-11b), 1.83 (1H, m, H-1b), 1.78 (1H, m, H-22b), 1.73 (1H, m, H-15b), 1.69 (1H, m, H-19b), 1.64 (1H, dd, H-9), 1.63 (1H, m, H-16a), 1.55–1.48 (2H, m, H-6), 1.52–1.59 (2H, m, H-7), 1.49 (1H, m, H-22a), 1.49 (1H, m, H-1a), 1.49 (1H, s, H-21b), 1.31 (1H, dd, H-5), 1.26 (1H, m, H-11a), 1.22 (1H, m, H-21a), 1.16 (1H, m, H-19a), 1.16 (3H, s, H-27), 1.14 (1H, m, H-15a), 1.09 (3H, s, H-23), 1.06 (3H, s, H-25), 1.04 (3H, s, H-24), 0.94 (3H, s, H-30), 0.92 (3H, s, H-29), 0.83 (3H, s, H-26); ^13^C NMR (CDCl_3_, 400 MHz): *δ* 217.61 (C-3), 182.99 (C-28), 143.64 (C-13), 122.43 (C-12), 55.36 (C-5), 47.43 (C-9), 46.88 (C-4), 46.58 (C-17), 45.85 (C-19), 41.79 (C-14), 41.13 (C-18), 39.31 (C-8), 39.13 (C-1), 36.81 (C-10), 34.14 (C-2), 33.83 (C-21), 33.04 (C-29), 32.41 (C-7), 32.21 (C-22), 30.67 (C-20), 27.70 (C-15), 26.47 (C-23), 25.81 (C-27), 23.55 (C-16), 23.51 (C-30), 22.97 (C-11), 21.44 (C-24), 19.58 (C-6), 16.96 (C-26), 15.01 (C-25).

### 3.4. Cell Culture and Viability Assay

Triple-negative human breast cancer cell line MDA-MB-231 was obtained from Istituto Scientifico Tumori (Genoa, Italy) and cultured as previously reported [38].

Before each experiment, cells were plated on 6 (1.5 × 10^5^ cells/2 mL) or 96 well plates (8 × 10^3^ cells/200 µL) and were allowed to adhere overnight before the treatment with sesquiterpene lactones or vehicles only. The determination of IC_50_ values was performed using Graphpad Prism 4.0 software (Graph PadPrismTM Software Inc., San Diego, CA, USA).

The morphological changes induced by treatments were evaluated using an OPTICA inverted microscope (OPTIKA S.r.l., Ponteranica, BG, Italy). The apoptotic cell morphology was also evaluated by acridine orange and ethidium bromide double staining, as reported by Liu et al. [39]. Stock solutions of compounds were prepared in dimethyl sulfoxide (DMSO) and diluted to final concentrations in DMEM. The concentrations of DMSO used as vehicle never exceeded 0.04% and did not exert toxic effects on MDA-MD-231 cells in comparison to the control. All the reagents used for the cell cultures were purchased from Biosigma (Cona, VE, Italy).

### 3.5. Assessment of Cell Viability

The effects of the compounds on cell viability were ascertained using the MTT (3-(4,5-Dimethylthiazol-2-yl)-2,5-Diphenyltetrazolium Bromide) colorimetric assay as previously described [38]. Cell viability was expressed as the percentage of the absorbance value of treated cells compared with untreated samples used as control. Each experiment was performed three times and analyzed for statistical significance.

### 3.6. Assessment of Autophagy by Monodansylcadaverine Test

The formation of autophagic vacuoles was ascertained by monodansylcadaverine (MDC) test as previously reported [40]. Three different visual fields were examined for each condition.

### 3.7. Apoptotic Cell Demise by Hoechst and Annexin V-FITC/PI Staining

The analysis of condensed or fragmented chromatin was assessed by vital Hoechst 33342 (Invitrogen; Thermo Fisher Scientific, Inc., Waltham, MA, USA ) staining. For these experiments cells were incubated for 30 min with Hoechst 33342 before the administration of SL-1. At the end of treatment, cells were analyzed under a fluorescence microscope (OPTIKA IM3FL4 fluorescence microscope, OPTIKA S.r.l, Ponteranica, BG, Italy) equipped with DAPI filter (excitation wavelength of 372 nm and emission wavelength of 456 nm).

The presence of apoptotic cells was ascertained by Annexin V-FITC kit (cat n. 130-092-052) from Miltenyi Biotec (Bergisch Gladbach, Germany) as reported [41]. The results presented in the figures are representative of three independent experiments. FlowJo v10 software (BD Biosciences 283 Franklin Lakes, NJ, USA) was used to analyze the data.

### 3.8. Western Blotting

Western blotting analyses were performed as previously reported [42]. Briefly, MDA-MB-231 cells (1.5 × 10^5^ cells/2 mL) treated with compounds were lysed at 4 °C with lysis buffer (1% NP-40, 0.1% SDS, and 0.5% sodium deoxycholate in PBS and supplemented with a protease inhibitor cocktail). Protein concentration was determined using the Bradford protein assay (Bio-Rad Laboratories S.r.l., Segrate, Milan, Italy). An equivalent amount of proteins (30 μg) were resolved by SDS-PAGE (Sodium Dodecyl Sulfate-PolyAcrylamide Gel Electrophoresis). The gels were then transferred onto a nitrocellulose membrane (Bio-Rad). Immunodetection was carried out by incubating the membranes with specific primary antibodies: caspase-3 (sc-65487) and PARP-1 (sc-53643) provided by Santa Cruz Biotechnology (Santa Cruz, CA, USA). Finally, immunoreactive signals, developed using HRP-conjugated secondary antibodies (Promega Italia s.r.l, Milan, Italy), were visualized with enhanced chemiluminescence (ECL) reagents (Cyanagen, Bologna, Italy) and captured with ChemiDoc XRS (Bio-Rad Laboratories, Inc., Hercules, CA, USA)). Signal quantification was performed using Quantity One 1-D softwarev 4.6.6 (Bio-Rad). Densitometry analysis of the bands was performed using Image J software v1.53K (https://imagej.net). Protein expression was normalized with *γ*-Tubulin (A5060; Sigma-Aldrich Merck KGaA)) as control.

### 3.9. Measurement of Intracellular ROS Content

Generation of intracellular ROS was estimated by the cell-permeant 2′,7′-dichlorodihydrofluorescein diacetate (H_2_DCFDA, Molecular Probes; Eugene, OR, USA), as previously described [40]. For these experiments, MDA-MB-231 cells (8 × 10^3^/200 µL) seeded in 96-well plates were treated with compound SL-1 for different times. Then, the medium was removed, and cells were incubated with a 5 μM H_2_DCFDA for 15 min at 37 °C in the dark. Fluorescence analyses were performed with an OPTIKA IM3FL4 fluorescence microscope (OPTIKA S.r.l, Ponteranica, BG, Italy) with a FITC filter (excitation wavelength of 485 nm and emission wavelength of 530 nm). Exposure times were set up and optimized to achieve minimal background noise. Representative pictures were selected and acquired by a digital imaging camera system (OPTIKA).

### 3.10. Evaluation of the Mitochondrial Membrane Potential

The mitochondrial membrane potential difference (ΔΨm) was measured by using the lipophilic cationic fluorochrome JC-1 (5,5′,6,6′-Tetrachloro-1,1′,3,3′-tetraethylbenzimidazolylcarbocyanine iodide, Invitrogen by Thermo Fisher Scientific, Inchinnan Business Park, Paisley, PA4 9RF, UK). Cells were seeded in 96-well plates (6 × 10^4^ cells/1 mL of medium) and, after treatment with the SL-1, they were incubated at 37 °C for 10 min with 5 µg/mL JC-1 following the manufacturer’s protocol. Then, the culture medium containing the fluorochrome was removed, and the cells were examined under a fluorescence microscope (OPTIKA IM3FL4 fluorescence microscope, OPTIKA S.r.l, Ponteranica, BG, Italy). Red fluorescence (J-aggregates), detected by a “rhodamine filter” with a 596 nm excitation peak polarized and have high membrane potential. Green fluorescence (monomeric form of JC-1), detected by a “FITC filter” with a 485 nm excitation peak and a 530 nm emission peak, is prevalent in apoptotic cells with a low membrane potential. Exposure times were set up and optimized to achieve minimal background noise. The images shown are the result of the overlay of the pictures captured with red and green fluorescence (OPTIKA PROVIEW, version x64, 4.11.20805.20220506).

### 3.11. Statistical Analysis

The results were represented as mean ± standard deviations (S.D.). Each experiment was performed in triplicate and repeated three times with similar results. Statistical analysis was performed using the GraphPadPrismTM 4.0 software (Graph PadPrismTM Software Inc., San Diego, CA, USA). Data was analyzed using Student’s t test and one-way ANOVA followed by the Bonferroni multiple comparisons test. Differences were considered significant when *p* < 0.05, and one-way ANOVA was used as the statistical method for data evaluation.

## 4. Conclusions

Guaianolide-type sesquiterpene lactones represent an interesting group of plant-derived compounds that possess enhanced inflammatory and anticancer properties [8,43,44,45,46], which render them promising anticancer drugs. The effects of guaianolide sesquiterpene lactones isolated from *Euphorbia microsphaera* Boiss. [43], *Carpesium faberi* [44], and Balkan endemic *Laserpitium* species [8] have been reported in estrogen-positive breast cancer cells showing antitumoral potential. However, no studies are reported on triple negative breast cancer cells, the most aggressive breast cancer cell type. In our study the phytochemical analysis of the roots of *L*. *siler* subsp. *siculum* led to the isolation of two sesquiterpene lactones, a mix of other two and one triterpenoid. Compounds SL-1 and 5 were already known, but their presence has not been reported in *Laserpitium* genus plants to date. To explore their anticancer potential, these compounds were evaluated in triple-negative breast cancer (TNBC) cells. These findings provide compelling evidence that all isolated sesquiterpene lactones exhibit significant cytotoxic activity, primarily through the induction of autophagy, oxidative stress, and mitochondrial impairment, ultimately committing TNBC cells to apoptotic cell death. Notably, SL-1 emerged as the most potent compound, displaying a marked ability to reduce cell viability in a dose- and time-dependent manner. While all SLs triggered autophagic vacuole formation at 24 h, prolonged exposure (48 h) led to a shift toward apoptosis, as confirmed by acridine orange/ethidium bromide staining and Western blot analyses of key apoptotic markers, including caspase-3 activation and PARP-1 cleavage. Further mechanistic investigations revealed that SL-1 induces oxidative stress, as evidenced by a significant increase in reactive oxygen species (ROS) production within the first phases of treatment. This oxidative burden was accompanied by upregulation of the antioxidant defense enzymes MnSOD and HO-1, suggesting an initial cellular attempt to counteract ROS-induced damage. However, sustained oxidative stress ultimately led to mitochondrial dysfunction, as indicated by JC-1 staining and the downregulation of VDAC-1, a crucial mitochondrial membrane protein involved in metabolic homeostasis. The loss of mitochondrial membrane potential, coupled with the biochemical markers of apoptosis, strongly supports the hypothesis that mitochondrial impairment plays a central role in SL-1-induced cytotoxicity. In conclusion, these findings highlight *L. siler* subsp. *siculum* as a promising source of bioactive compounds with potent anticancer properties. The ability of its sesquiterpene lactone SL-1 to induce oxidative stress and disrupt mitochondrial integrity provides valuable insights into its mechanism of action, opening new avenues for the development of targeted therapies against aggressive breast cancer subtypes. Future studies should better focus on the molecular pathways involved and evaluating the therapeutic potential of these bioactive phytocompounds in in vivo models to assess their efficacy and safety in a physiological context.

## Figures and Tables

**Table 1:** IC_50_ values of *Laserpitium siler* sesquiterpene lactones SL-1, SL-2 and SL-3–4 against triple negative MDA-MB-231 cells.

IC_50_ (μΜ)		
SL-1	SL-2	SL-3–4
50 ± 5	90 ± 3	250 ± 2

## Data Availability

Dataset available on request from the authors.

## Supplementary Materials

The following supporting information can be downloaded at: https://www.mdpi.com/article/10.3390/plants14213289/s1, Figure S1. Structure and NMR data (400 MHz, CDCl_3_) of SL-1. Figure S2. Structure and NMR data (400 MHz, CDCl_3_) of SL-2. Figure S3. Structure and NMR data (400 MHz, CDCl_3_) of SL-3. Figure S4. Structure and NMR data (400 MHz, CDCl_3_) of SL-4. Figure S5. Structure and NMR data (400 MHz, CDCl_3_) of 5. Figure S6. Effects of sesquiterpene lactones on MDA-MB-231 breast cancer cell morphology. Figure S7. 3D structure of SL-2 and main NOESY correlations. Figure S8. NMR spectra of SL-1 (Panel Aà ^1^H-NMR, Panel B à ^13^C-NMR) 400 MHz, CDCl_3_. Figure S9. NMR spectra of SL-2 (Panel Aà ^1^H-NMR, Panel B à ^13^C-NMR, Panel C à ^1^H-^1^H COSY, Panel D àHSQC, Panel E à HMBC, Panel F à NOESY) 400 MHz, CDCl_3_. Figure S10. NMR spectra of SL-3-4 (Panel Aà ^1^H-NMR, Panel B à ^13^C-NMR, Panel C à ^1^H-^1^H COSY, Panel D àHSQC, Panel E à HMBC, Panel F à NOESY) 400 MHz, CDCl_3_. Figure S11. NMR spectra of 5 (Panel Aà ^1^H-NMR, Panel B à ^13^C-NMR) 400 MHz, CDCl_3_.Figure S12. HRESI-MS spectrum of SL-1 (positive ion mode). Figure S13. HRESI-MS spectrum of SL-2 (positive ion mode). Figure S14. HRESI-MS spectrum of SL-3 (positive ion mode). Figure S15. HRESI-MS spectrum of SL-4 (positive ion mode).
