# Supplementary material for: Exploring the Biochemical Mechanism Beyond the Cytotoxic Activity of Sesquiterpene Lactones from Sicilian Accession of Laserpitium siler Subsp. siculum (Spreng.) Thell
Source: Plants (Basel). 2025 Oct 28;14(21):3289. doi: 10.3390/plants14213289 (PMC12608240; doi:10.3390/plants14213289)
Supplement: Supplementary file 1 [file plants-14-03289-s001.zip › plants-3920938-supplementary.pdf]

SUPPLEMENTARY MATERIAL

Exploring the biochemical mechanism beyond the cytotoxic activity of sesquiterpene lactones from Sicilian accession of *Laserpitium siler* subsp. *siculum* (Spreng.) Thell.

Alessandro Vaglica [1], Antonella Maggio [1,*], Chiara Occhipinti [1], Natale Badalamenti [1], Marianna Lauricella [2], Maurizio Bruno [1] and Antonella D'Anneo [1]

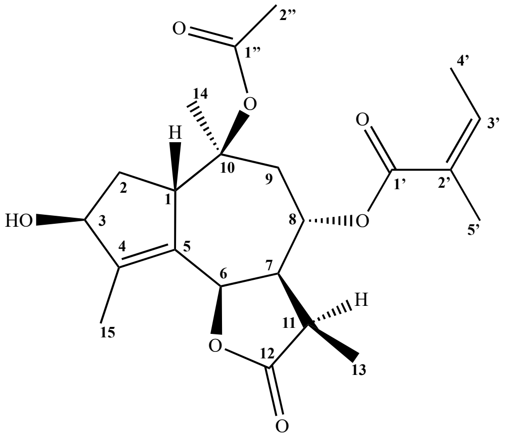

SL-1

Position	δ_C, type	δ_H (*J* in Hz)
**1**	49.98, CH	3.27, br d (8.3)
**2**	37.95, CH_2	α = 1.70, ddd (13.8, 8.3, 6.8)<br>β = 2.59, dd (13.8, 6.4)
**3**	79.31, CH	4.77, dd (6.8, 6.4)
**4**	148.21, C	-
**5**	131.64, C	-
**6**	75.12, CH	5.34, d (6.3)
**7**	48.47, CH	2.80, ddd (9.6, 7.6, 6.3)
**8**	66.28, CH	5.23, dd (9.6, 9.6)
**9**	43.82, CH_2	α = 1.77, dd (15.4, 9.6)<br>β = 2.66, d (15.4)
**10**	83.97, C	-
**11**	39.92, CH	2.99, dq (7.6)
**12**	177.98, C	-
**13**	11.28, CH_3	1.22, d (7.6)
**14**	21.64, CH_3	1.39, s
**15**	11.21, CH_3	1.86, m
**1′**	166.14, C	-
**2′**	127.37, C	-
**3′**	138.90, CH	6.05, qq (7.2, 1.3)
**4′**	15.64, CH_3	1.96, dq (7.2, 1.3)
**5′**	20.31, CH_3	1.84, dq (1.3, 1.3)
**1′′**	171.4, C	-
**2′′**	22.61, CH_3	2.09, s

Figure S1. Structure and NMR data (400 MHz, $CDCl_3$) of **SL-1.**

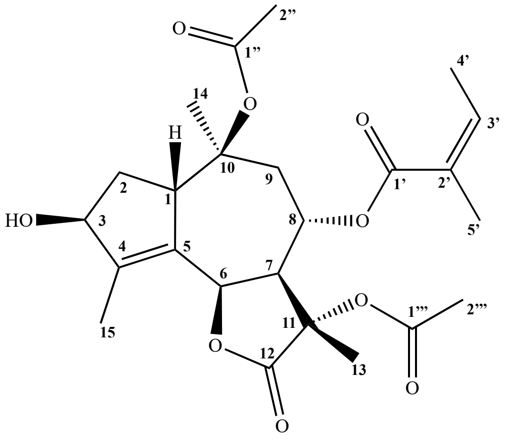

SL-2

Position	δC, type	δH (*J* in Hz)
**1**	52.46, CH	3.18, br d (8.3)
**2**	36.46, CH_2	α = 1.73, ddd (14.1, 8.3, 7.1)<br>β = 2.52, dd (14.1, 6.4)
**3**	79.69, CH	4.74, dd (7.1, 6.4)
**4**	150.61, C	-
**5**	130.90, C	-
**6**	73.15, CH	5.64, d (10.5)
**7**	48.99, CH	3.54, dd (10.5, 10.5)
**8**	66.19, CH	5.50, dd (10.5, 10.5)
**9**	40.62, CH_2	α = 1.50, dd (15.6, 10.5)<br>β = 2.90, d (15.6)
**10**	83.50, C	-
**11**	79.19, C	-
**12**	173.96, C	-
**13**	20.75, CH_3	1.57, s
**14**	20.20, CH_3	1.31, s
**15**	12.16, CH_3	1.93, brs
**1′**	166.23, C	-
**2′**	127.13, C	-
**3′**	139.44, CH	6.12, qq (7.2, 1.3)
**4′**	15.71, CH_3	1.98, dq (7.2, 1.3)
**5′**	20.29, CH_3	1.85, dq (1.3, 1.3)
**1″**	178.98, C	-
**2″**	20.83, CH_3	2.08, s
**1‴**	170.10, C	-
**2‴**	22.56, CH_3	2.12, s

Figure S2. Structure and NMR data (400 MHz, $CDCl_3$) of **SL-2.**

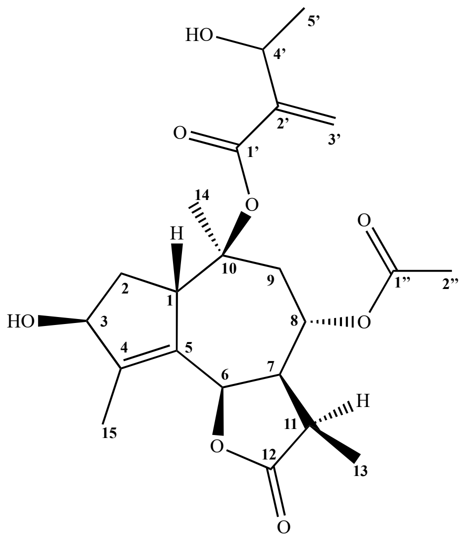

SL-3

Position	δC, type	δH (*J* in Hz)
**1**	49.82, CH	3.24, br d (8.3)
**2**	37.97, CH_2	α = 1.70, ddd (13.8, 8.3, 6.8)<br>β = 2.62, dd (13.8, 6.4)
**3**	79.22, CH	4.78, dd (6.8, 6.4)
**4**	148.48, C	-
**5**	131.38, C	-
**6**	74.94, CH	5.35, d (6.3)
**7**	48.51, CH	2.84, ddd (9.6, 7.6, 6.3)
**8**	67.36, CH	5.25, dd (9.6, 9.6)
**9**	43.81, CH_2	α = 1.73, dd (15.6, 9.6)<br>β = 2.68, d (15.6)
**10**	83.81, C	-
**11**	39.92, CH	3.00, dq (7.6)
**12**	177.78, C	-
**13**	11.22, CH_3	1.20, d (7.6)
**14**	21.63, CH_3	1.39, s
**15**	11.22, CH_3	1.87, m
**1′**	164.96, C	-
**2′**	143.97, C	-
**3′**	123.81, CH_2	a = 6.10, d (1.1)<br>b = 5.85, d (1.1)
**4′**	67.04, CH	4.60, brq (6.5)
**5′**	22.62, CH_3	1.36, d (6.5)
**1′′**	170.97, C	-
**2′′**	22.20, CH_3	2.08, s

Figure S3. Structure and NMR data (400 MHz, $CDCl_3$) of **SL-3.**

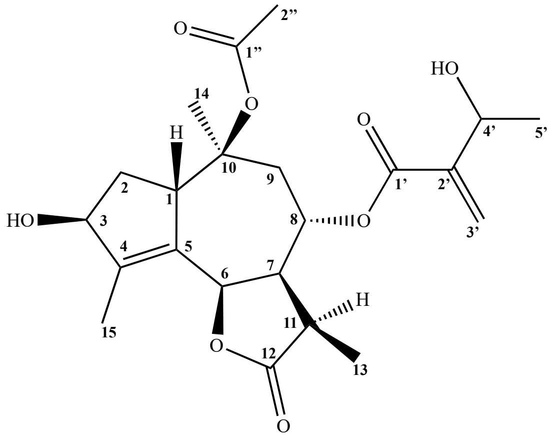

SL-4

Position	δC, type	δH (*J* in Hz)
**1**	49.82, CH	3.24, br d (8.3)
**2**	37.97, CH_2	α = 1.70, ddd (13.8, 8.3, 6.8)<br>β = 2.62, dd (13.8, 6.4)
**3**	79.22, CH	4.78, dd (6.8, 6.4)
**4**	148.54, C	-
**5**	131.38, C	-
**6**	74.94, CH	5.35, d (6.3)
**7**	48.51, CH	2.84, ddd (9.6, 7.6, 6.3)
**8**	67.21, CH	5.26, dd (9.6, 9.6)
**9**	43.71, CH_2	α = 1.75, dd (15.6, 9.6)<br>β = 2.66, d (15.6)
**10**	83.81, C	-
**11**	39.92, CH	3.00, dq (7.6)
**12**	177.78, C	-
**13**	11.22, CH_3	1.21, d (7.6)
**14**	21.63, CH_3	1.39, s
**15**	11.22, CH_3	1.87, m
**1′**	165.02, C	-
**2′**	143.97, C	-
**3′**	123.93, CH_2	a = 6.11, d (1.1)<br>b = 5.85, d (1.1)
**4′**	67.04, CH	4.60, brq (6.5)
**5′**	22.58, CH_3	1.37, d (6.5)
**1′′**	170.90, C	-
**2′′**	22.20, CH_3	2.08, s

Figure S4. Structure and NMR data (400 MHz, $CDCl_3$) of **SL-4**

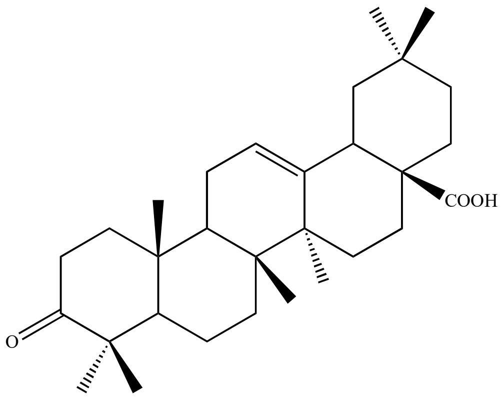

5

Position	δC, type	δH (*J* in Hz)
**1**	39.13, CH_2	a = 1.49, m<br>b = 1.83, m
**2**	34.14, CH_2	2.54-2.37, m
**3**	217.61, C	-
**4**	46.88, C	-
**5**	55.36, CH	1.31, dd
**6**	19.58, CH_2	1.55-1.48, m
**7**	32.41, CH_2	1.52-1.59, m
**8**	39.31, C	-
**9**	47.43, CH	1.64, dd
**10**	36.81, C	-
**11**	22.97, CH_2	a = 1.26, m<br>b = 1.99, m
**12**	122.43, CH	5.29, t
**13**	143.64, C	-
**14**	41.79, C	-
**15**	27.70, CH_2	a = 1.14, m<br>b = 1.73, m
**16**	23.55, CH_2	a = 1.63, m<br>b = 1.99, m
**17**	46.58, C	-
**18**	41.13, CH	2.85, dd (11.4)
**19**	45.85, CH_2	a = 1.16, m<br>b = 1.65, m
**20**	30.67, C	-
**21**	33.83, CH_2	a = 1.22, m<br>b = 1.49, m
**22**	32.21, CH_2	a = 1.49, m<br>b = 1.78, m
**23**	26.47, CH_3	1.09, s
**24**	21.44, CH_3	1.04, s
**25**	15.01, CH_3	1.06, s
**26**	16.96, CH_3	0.83, s
**27**	25.81, CH_3	1.16, s
**28**	182.99, C	-
**29**	33.04, CH_3	0.92, s
**30**	23.51, CH_3	0.94, s

Figure S5. Structure and NMR data (400 MHz, $CDCl_3$) of **5.**

Panel A

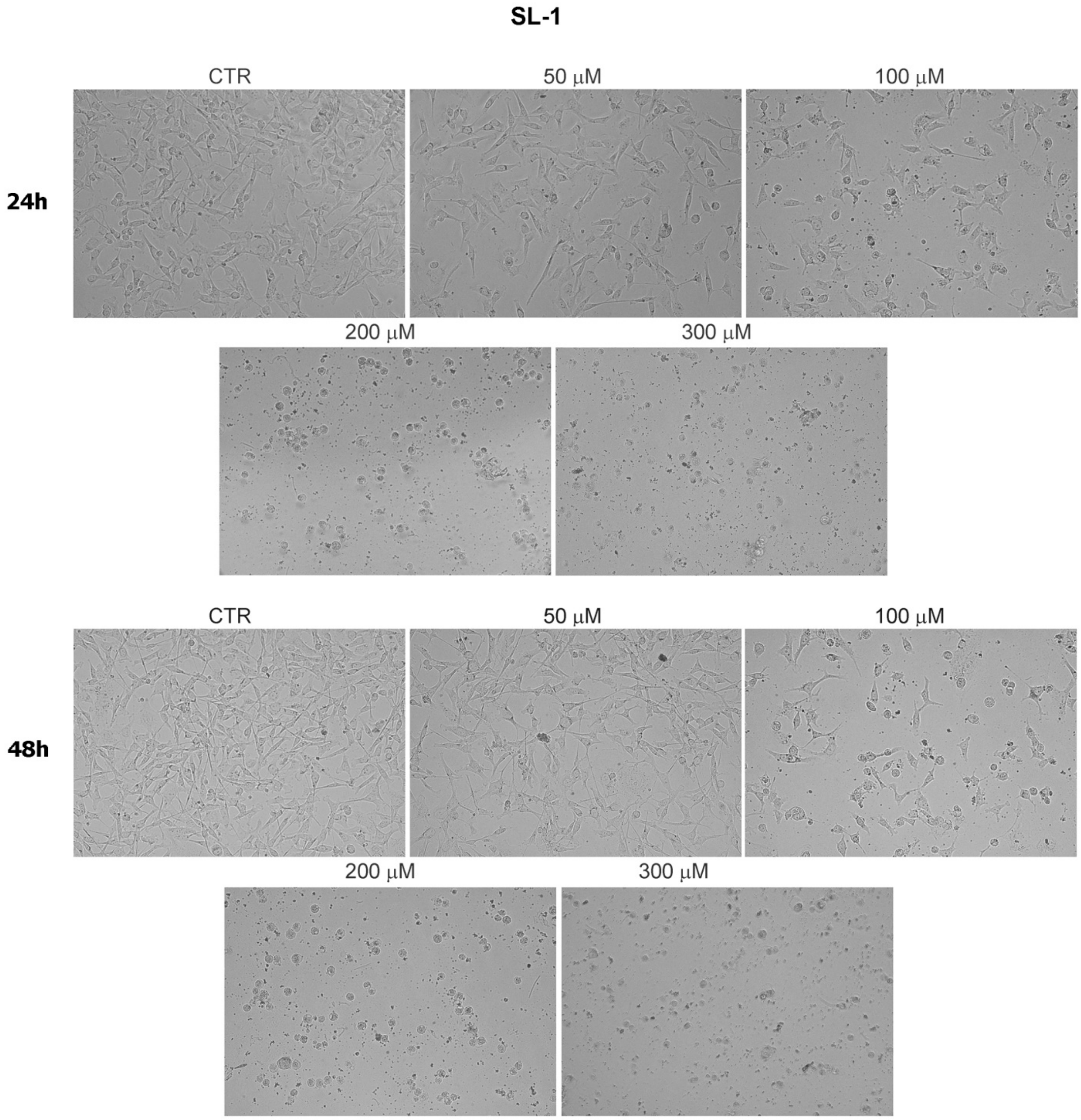
SL-1
CTR
50 μM
100 μM
24h
200 μM
300 μM
CTR
50 μM
100 μM
48h
200 μM
300 μM

Panel B

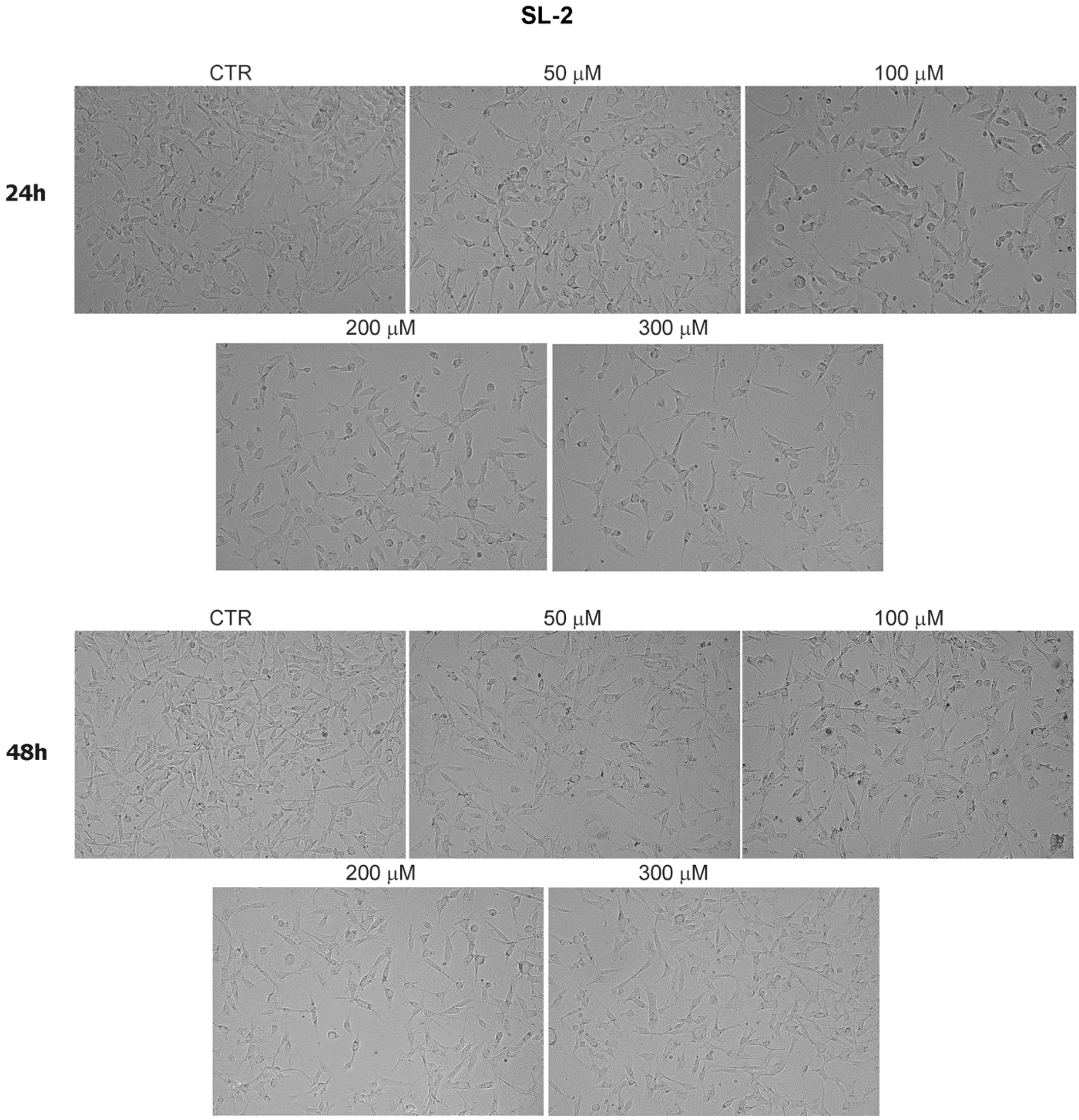
SL-2
CTR
50 μM
100 μM
24h
200 μM
300 μM
CTR
50 μM
100 μM
48h
200 μM
300 μM

Panel C

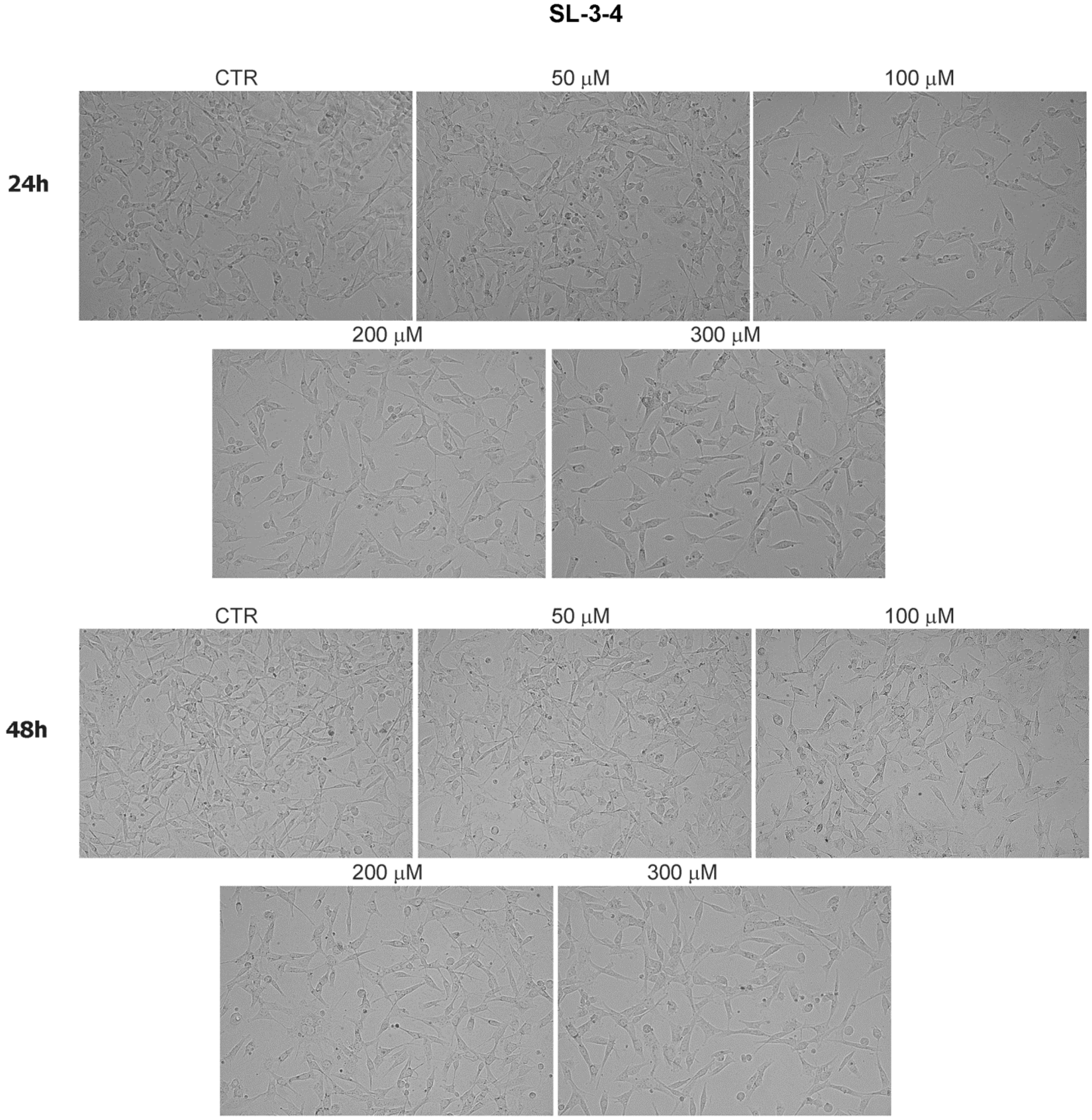

Figure S6. Effects of sesquiterpene lactones on MDA-MB-231 breast cancer cell morphology. Cells were seeded on 96 well plates (8x10^3) and exposed to treatment with compound **SL-1** (panel A), **SL-2** (panel B), and **SL-3-4** (panel C), for 24h and 48h, respectively. Pictures were taken at 200X magnification using an inverted OPTIKA IM3FL4 microscope equipped with OPTIKA PROVIEW imaging system (OPTIKA Srl, Italia).

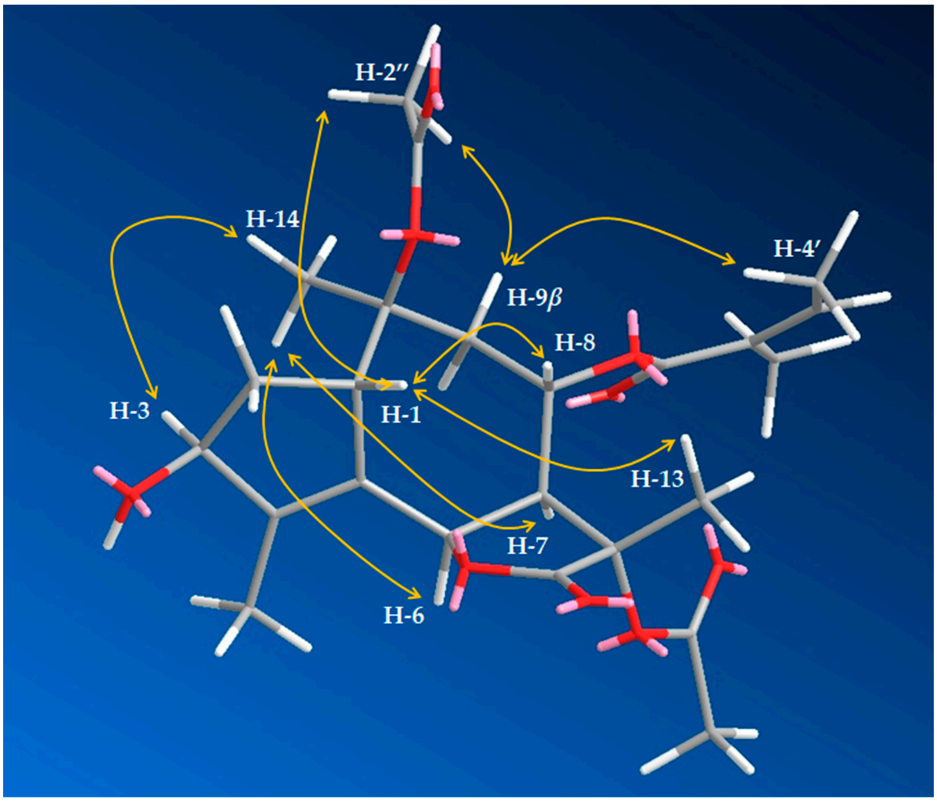

Figure S7. 3D structure of SL-2 and main NOESY correlations.

Panel A

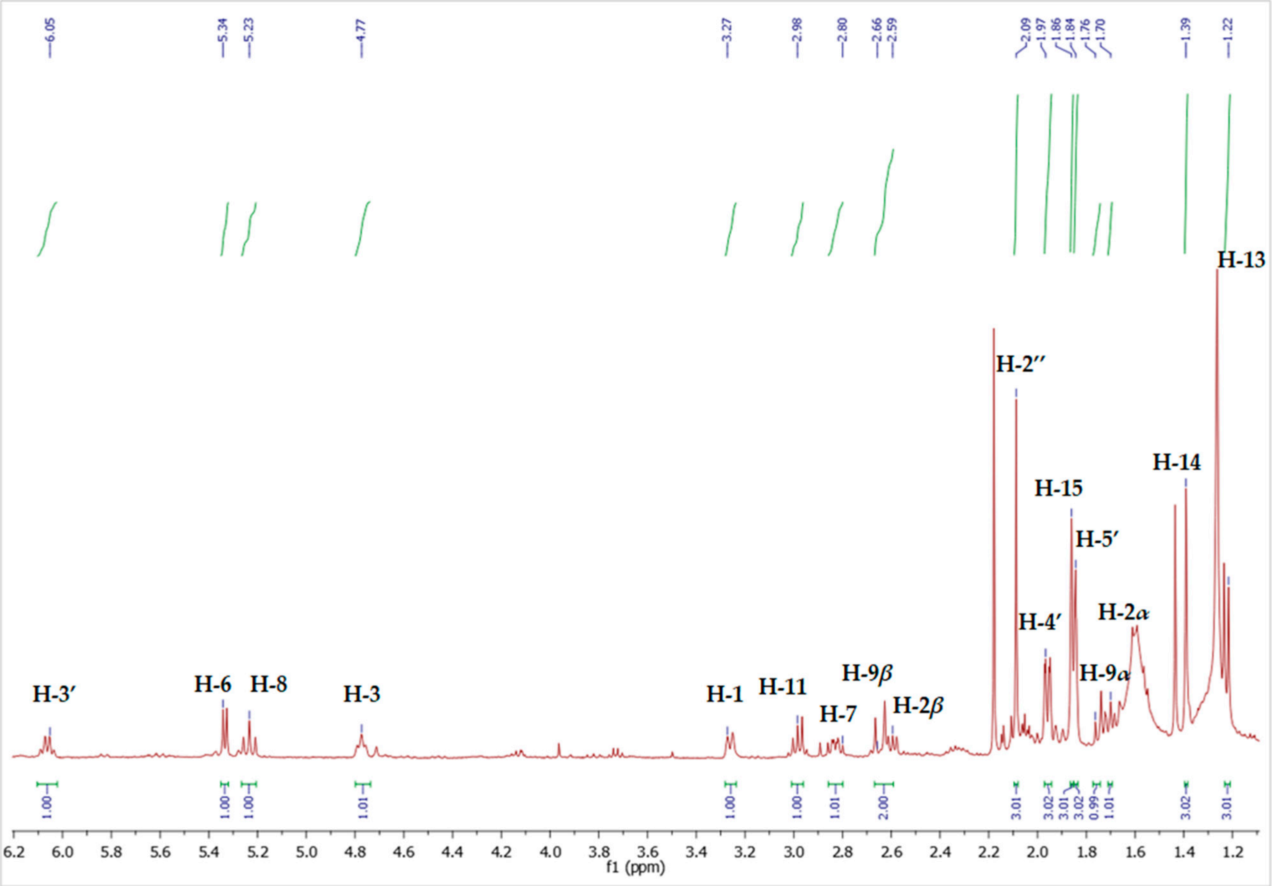
6.05
5.34
5.23
4.77
3.27
2.98
2.80
2.66
2.59
2.09
1.97
1.86
1.84
1.76
1.70
1.39
1.22
H-13
H-2″
H-14
H-15
H-5′
H-2α
H-4′
H-9β
H-9α
H-3′
H-6
H-8
H-3
H-1
H-11
H-7
H-2β
1.00
1.00
1.00
1.01
1.00
1.00
1.01
2.00
3.01
3.02
3.01
3.02
0.99
1.01
3.02
3.01
6.2 6.0 5.8 5.6 5.4 5.2 5.0 4.8 4.6 4.4 4.2 4.0 3.8 3.6 3.4 3.2 3.0 2.8 2.6 2.4 2.2 2.0 1.8 1.6 1.4 1.2
f1 (ppm)

Panel B

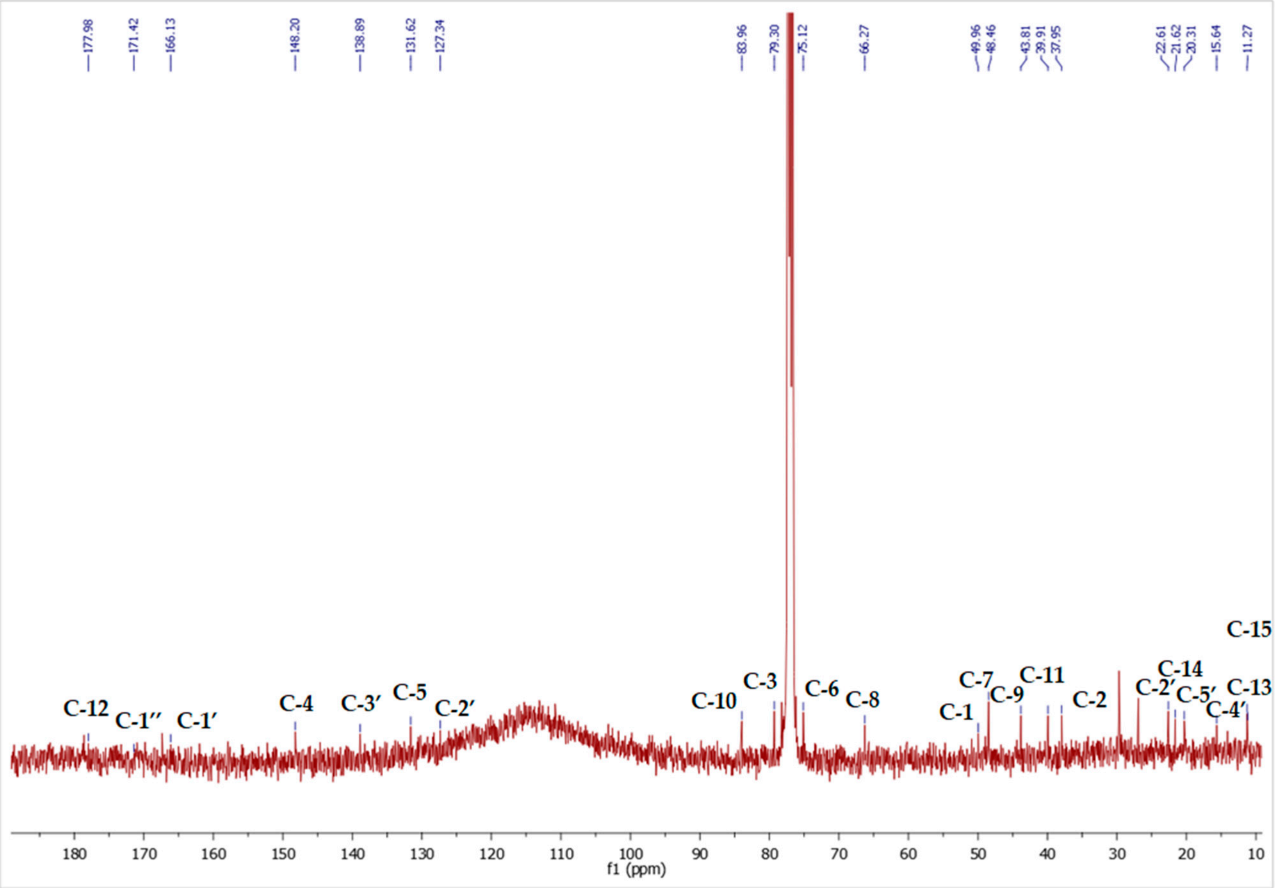

Figure S8. NMR spectra of **SL-1** (Panel A→ ^{1}H-NMR, Panel B → ^{13}C-NMR) 400 MHz, $CDCl_3$.

Panel A

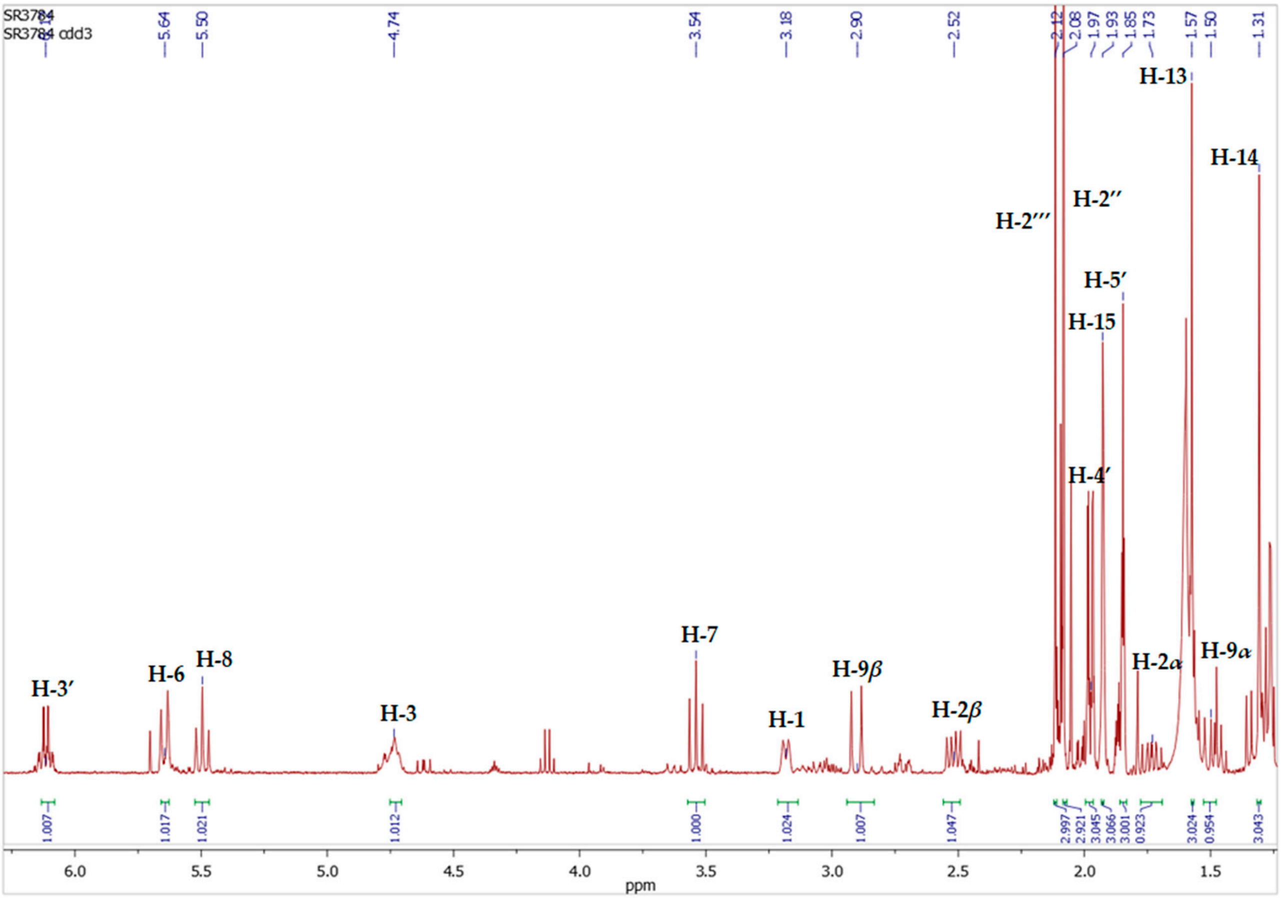

SR3784
SR3784 cdd3
H-3′
H-6
H-8
H-3
H-7
H-1
H-9β
H-2β
H-2‴
H-2″
H-5′
H-15
H-4′
H-13
H-14
H-2α
H-9α
ppm

Panel B

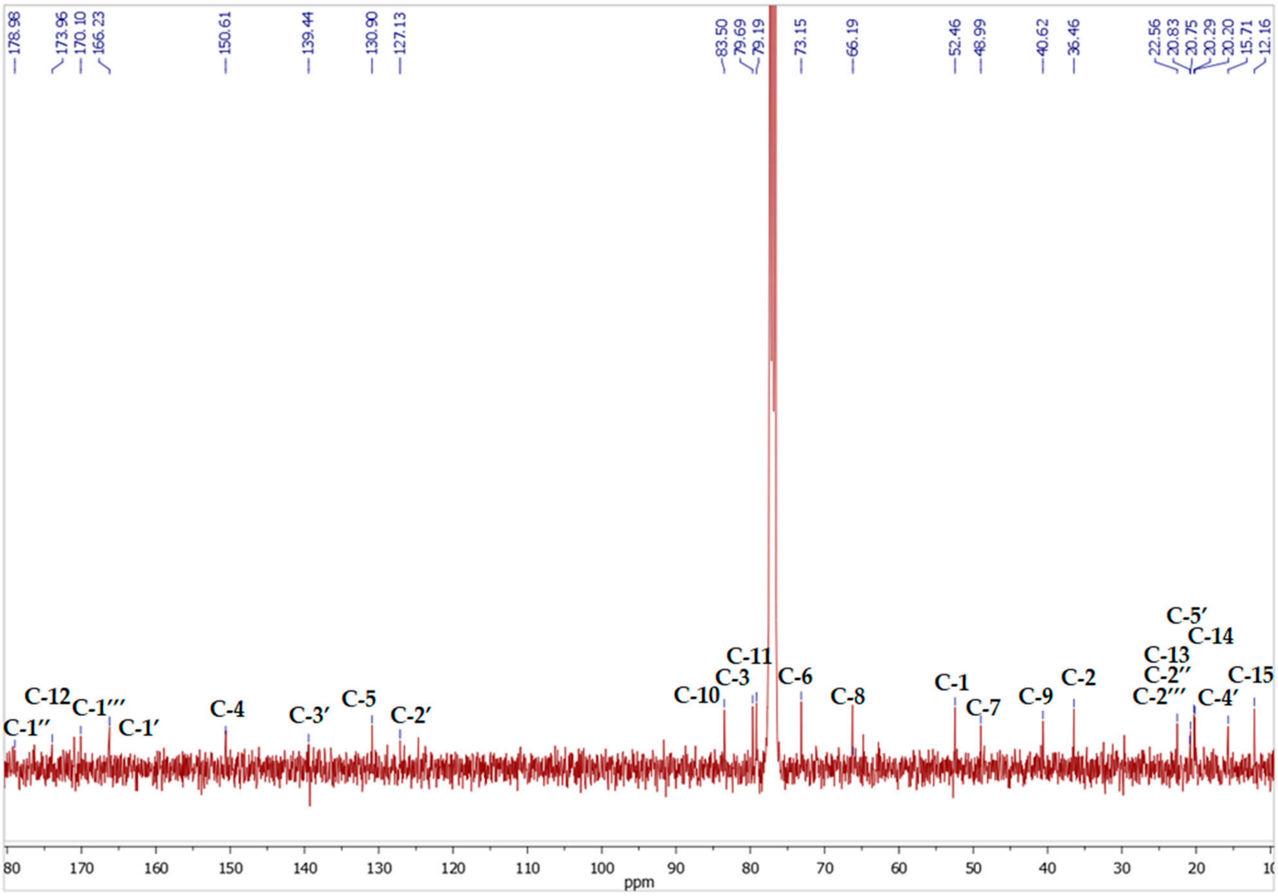
178.98
173.96
170.10
166.23
150.61
139.44
130.90
127.13
83.50
79.69
79.19
73.15
66.19
52.46
48.99
40.62
36.46
22.56
20.83
20.75
20.29
20.20
15.71
12.16
C-1″
C-12
C-1‴
C-1′
C-4
C-3′
C-5
C-2′
C-10
C-3
C-11
C-6
C-8
C-1
C-7
C-9
C-2
C-2‴
C-2″
C-13
C-5′
C-14
C-4′
C-15
ppm

Panel C

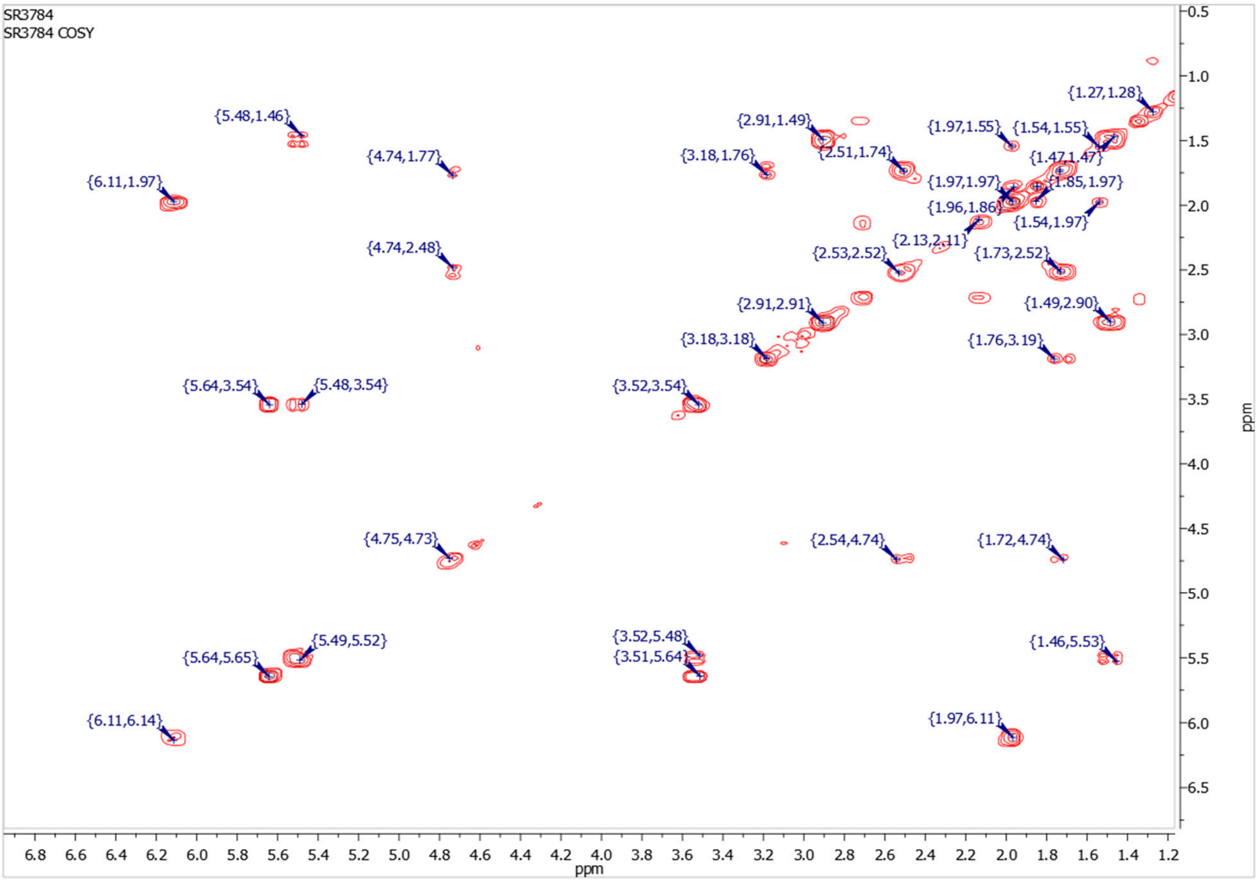

SR3784
SR3784 COSY
{5.48,1.46}
{6.11,1.97}
{4.74,1.77}
{4.74,2.48}
{5.64,3.54}
{5.48,3.54}
{4.75,4.73}
{5.64,5.65}
{5.49,5.52}
{6.11,6.14}
{2.91,1.49}
{3.18,1.76}
{2.51,1.74}
{2.53,2.52}
{2.91,2.91}
{3.18,3.18}
{3.52,3.54}
{2.54,4.74}
{3.52,5.48}
{3.51,5.64}
{1.27,1.28}
{1.97,1.55}
{1.54,1.55}
{1.47,1.47}
{1.97,1.97}
{1.85,1.97}
{1.96,1.86}
{1.54,1.97}
{2.13,2.11}
{1.73,2.52}
{1.49,2.90}
{1.76,3.19}
{1.72,4.74}
{1.46,5.53}
{1.97,6.11}
ppm

Panel D

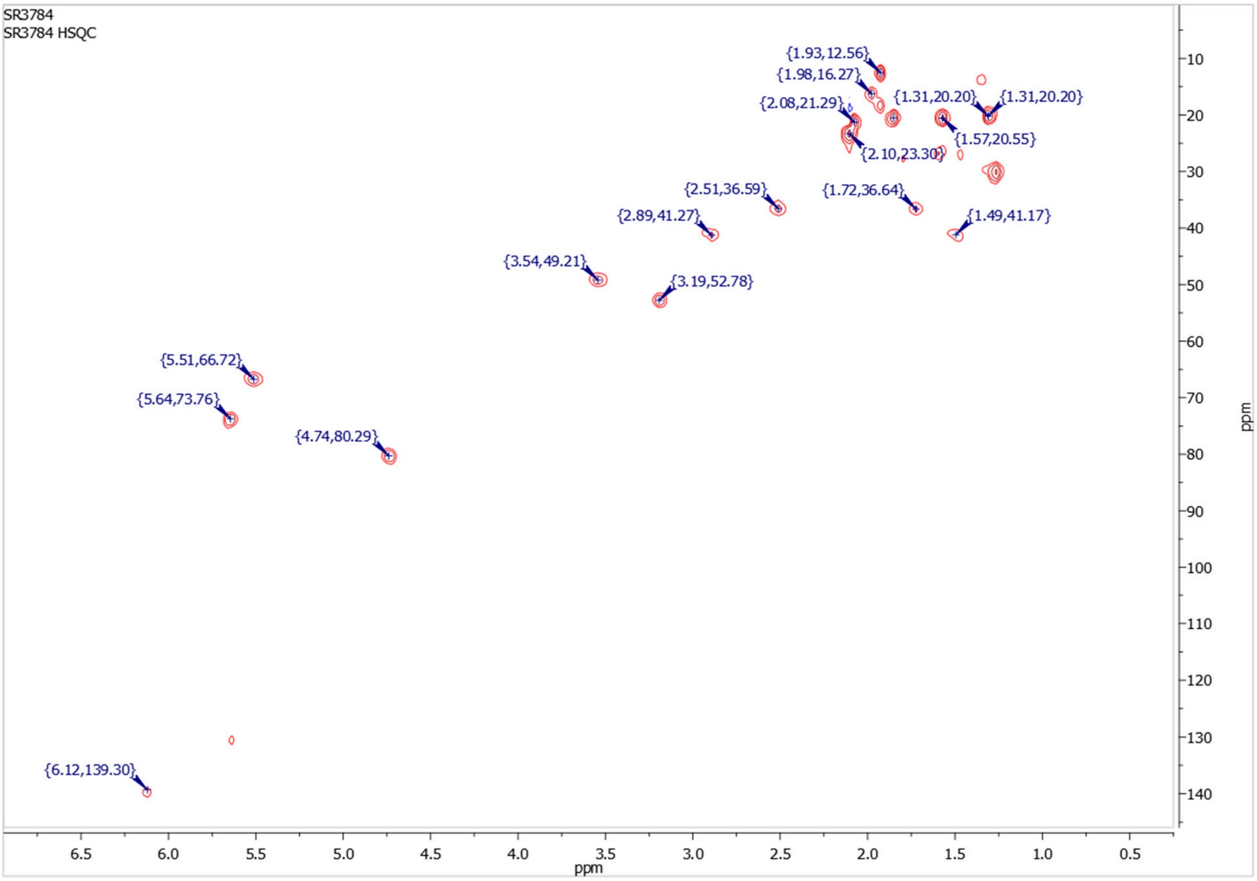

SR3784
SR3784 HSQC
{1.93,12.56}
{1.98,16.27}
{1.31,20.20}
{1.31,20.20}
{2.08,21.29}
{1.57,20.55}
{2.10,23.30}
{2.51,36.59}
{1.72,36.64}
{2.89,41.27}
{1.49,41.17}
{3.54,49.21}
{3.19,52.78}
{5.51,66.72}
{5.64,73.76}
{4.74,80.29}
{6.12,139.30}
ppm
ppm

Panel E

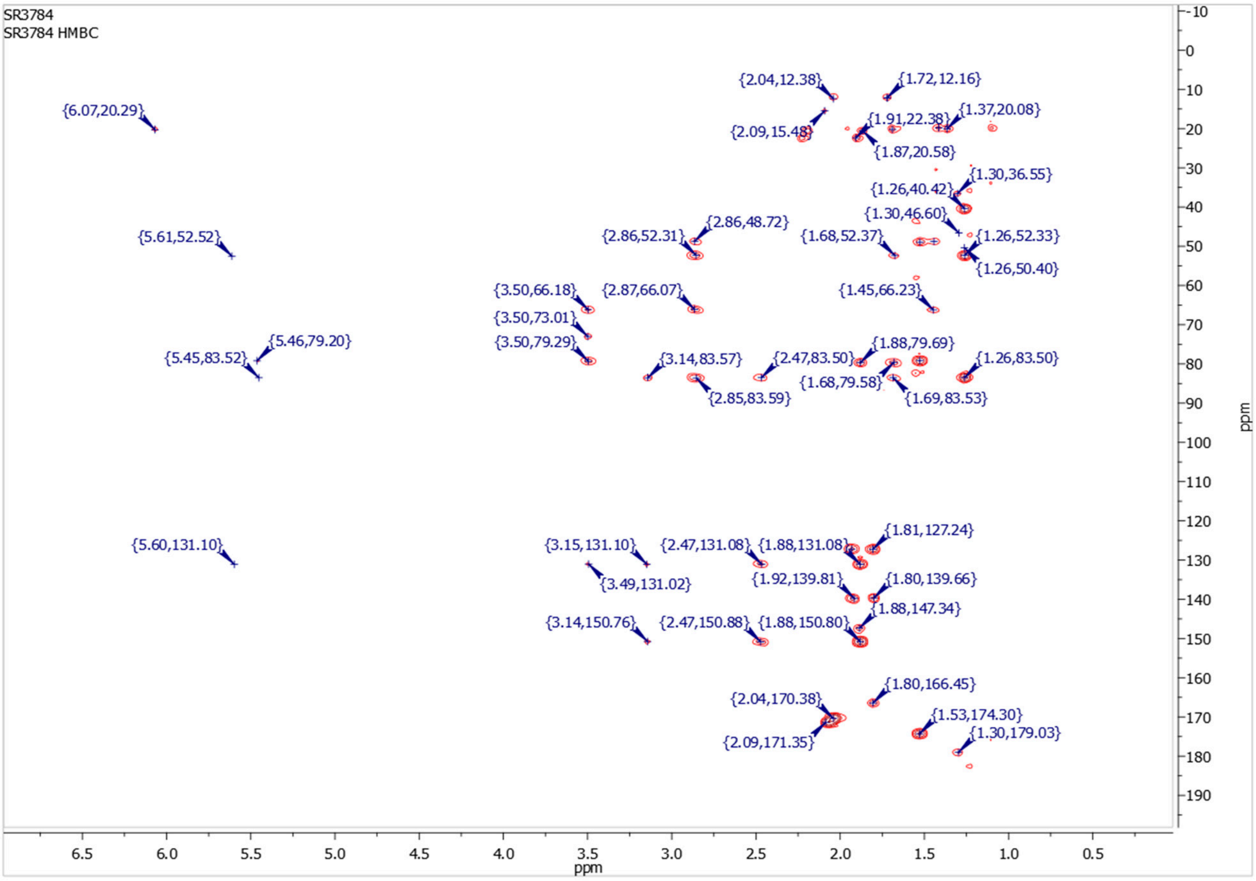
SR3784
SR3784 HMBC
{6.07,20.29}
{5.61,52.52}
{5.45,83.52}
{5.46,79.20}
{5.60,131.10}
{2.04,12.38}
{2.09,15.48}
{1.72,12.16}
{1.91,22.38}
{1.37,20.08}
{1.87,20.58}
{1.30,36.55}
{1.26,40.42}
{1.30,46.60}
{2.86,48.72}
{2.86,52.31}
{1.68,52.37}
{1.26,52.33}
{1.26,50.40}
{3.50,66.18}
{2.87,66.07}
{1.45,66.23}
{3.50,73.01}
{3.50,79.29}
{3.14,83.57}
{2.47,83.50}
{1.88,79.69}
{1.26,83.50}
{1.68,79.58}
{2.85,83.59}
{1.69,83.53}
{3.15,131.10}
{2.47,131.08}
{1.88,131.08}
{1.81,127.24}
{3.49,131.02}
{1.92,139.81}
{1.80,139.66}
{1.88,147.34}
{3.14,150.76}
{2.47,150.88}
{1.88,150.80}
{1.80,166.45}
{2.04,170.38}
{1.53,174.30}
{1.30,179.03}
{2.09,171.35}
ppm
ppm

Panel F

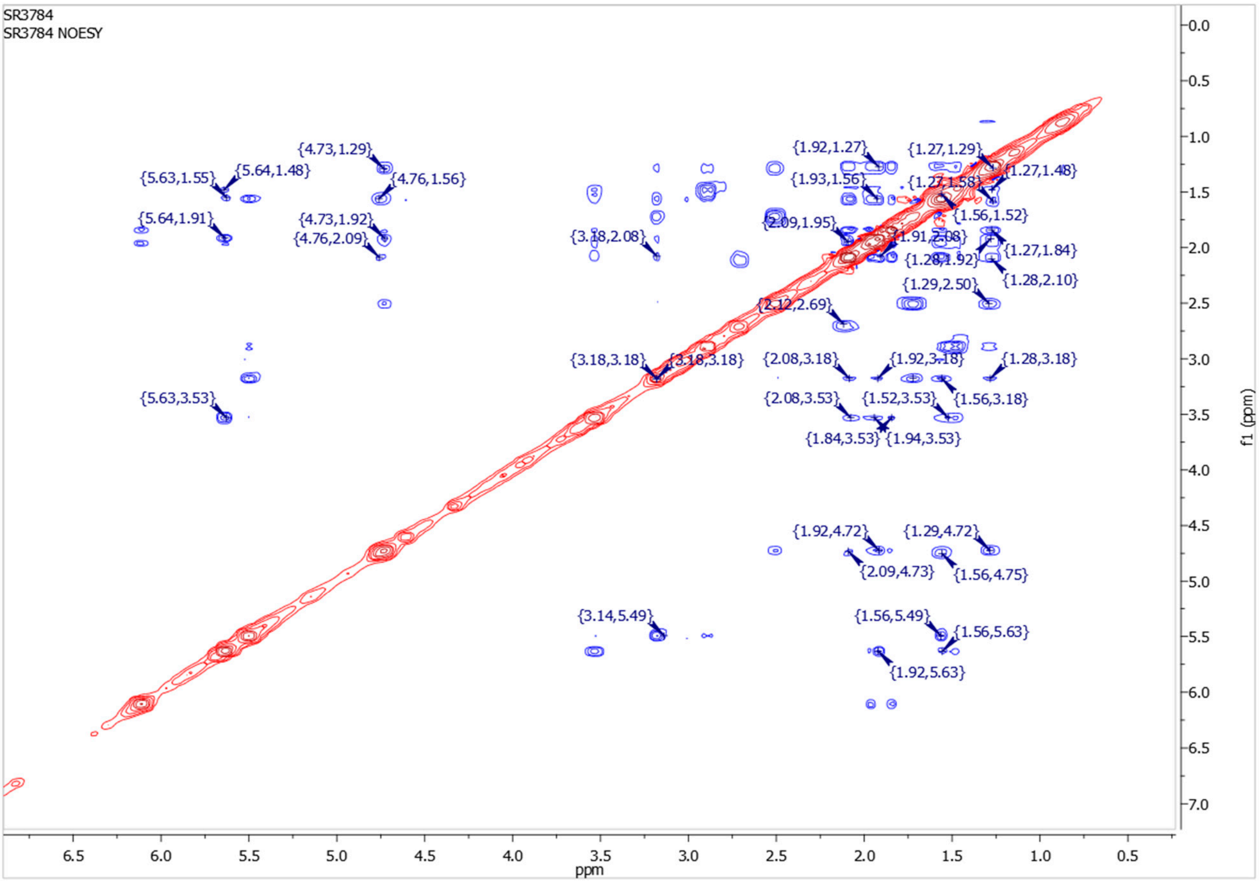

Figure S9. NMR spectra of **SL-2** (Panel A→ ^{1}H-NMR, Panel B → ^{13}C-NMR, Panel C → ^{1}H-^{1}H COSY, Panel D →HSQC, Panel E → HMBC, Panel F → NOESY) 400 MHz, $CDCl_3$.

Panel A

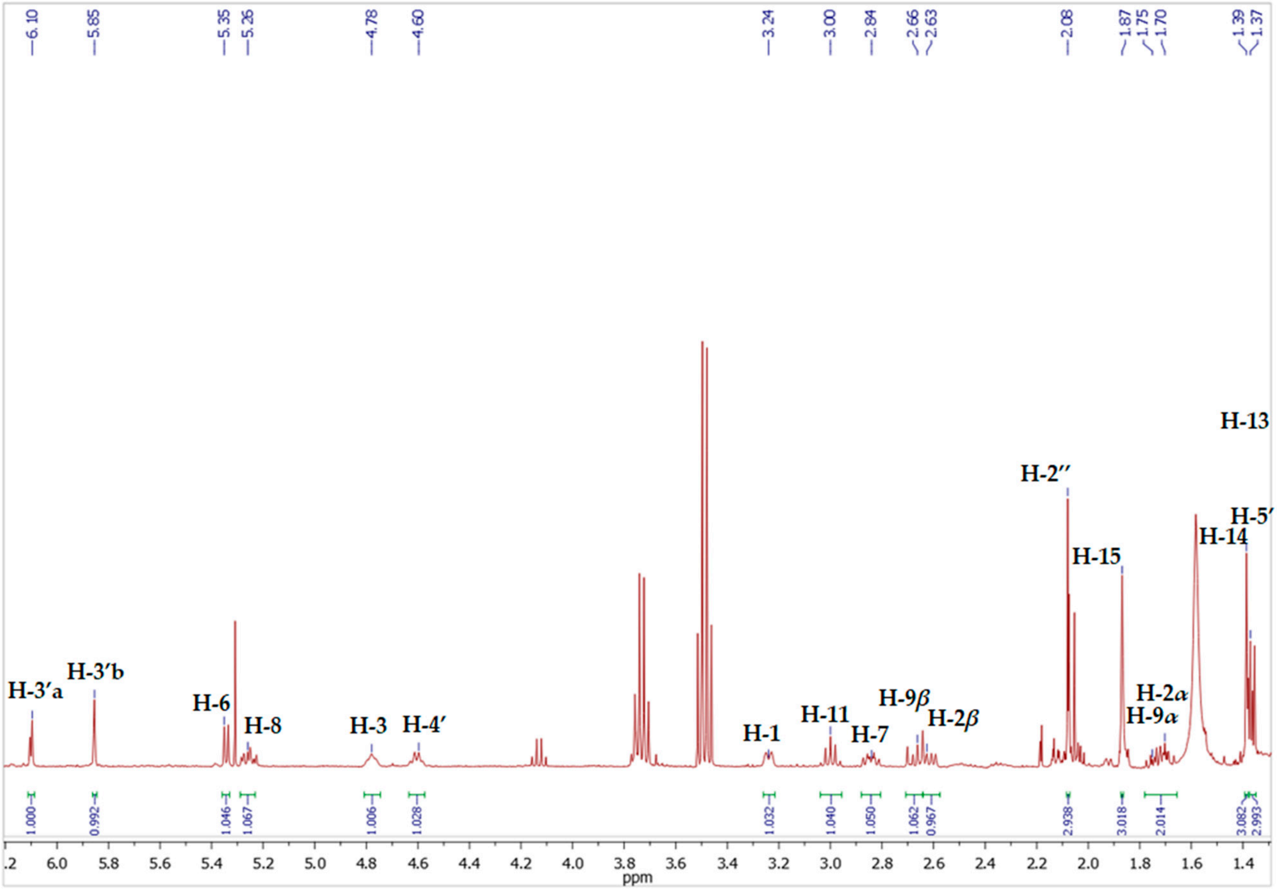

6.10
5.85
5.35
5.26
4.78
4.60
3.24
3.00
2.84
2.66
2.63
2.08
1.87
1.75
1.70
1.39
1.37
H-13
H-2′′
H-5′
H-14
H-15
H-3′a
H-3′b
H-6
H-8
H-3
H-4′
H-1
H-11
H-7
H-9β
H-2β
H-2α
H-9α
1.000
0.992
1.046
1.067
1.006
1.028
1.032
1.040
1.050
1.062
0.967
2.938
3.018
2.014
3.082
2.993
ppm

Panel B

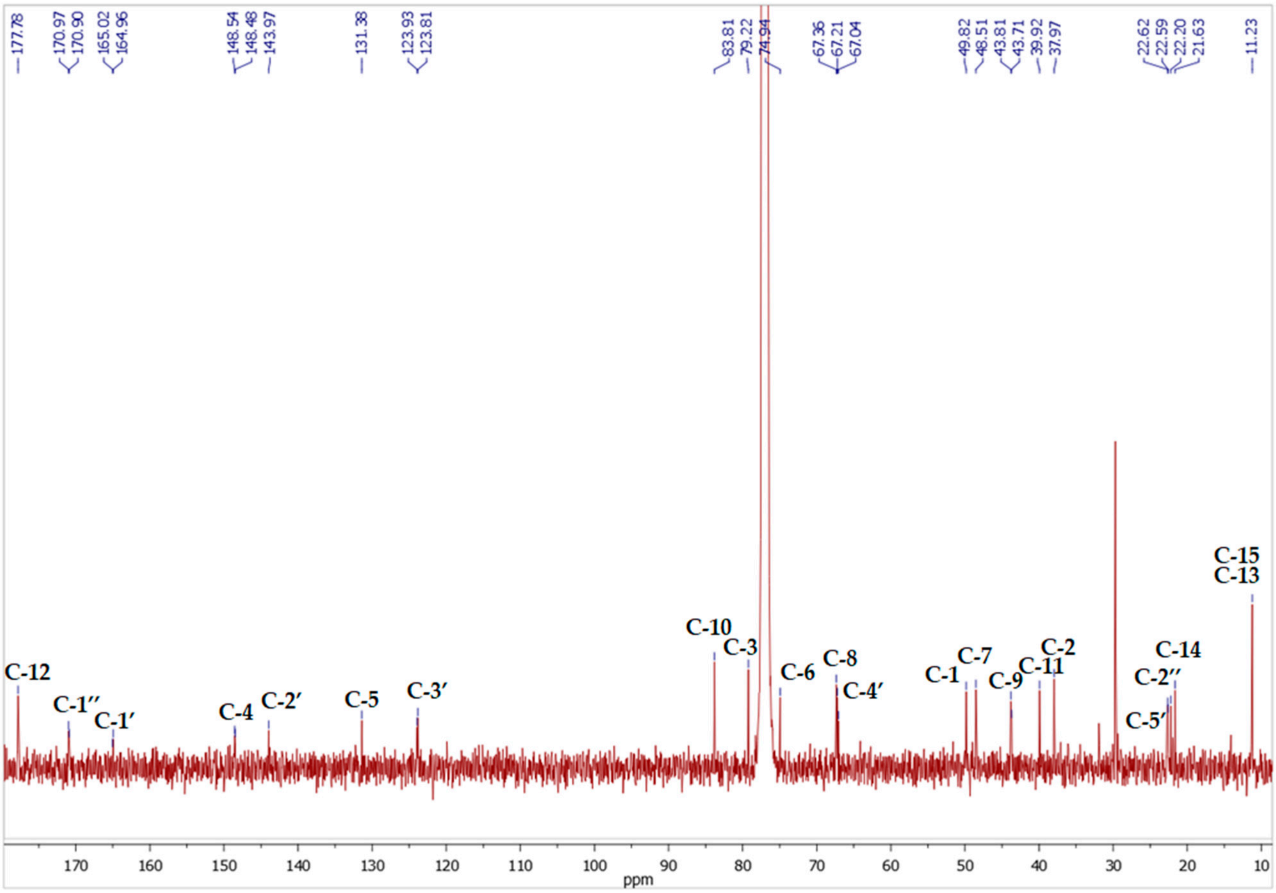
177.78
170.97
170.90
165.02
164.96
148.54
148.48
143.97
131.38
123.93
123.81
83.81
79.22
74.94
67.36
67.21
67.04
49.82
48.51
43.81
43.71
39.92
37.97
22.62
22.59
22.20
21.63
11.23
C-12
C-1''
C-1'
C-4
C-2'
C-5
C-3'
C-10
C-3
C-6
C-8
C-4'
C-1
C-7
C-9
C-11
C-2
C-5'
C-2''
C-14
C-15
C-13
ppm

Panel C

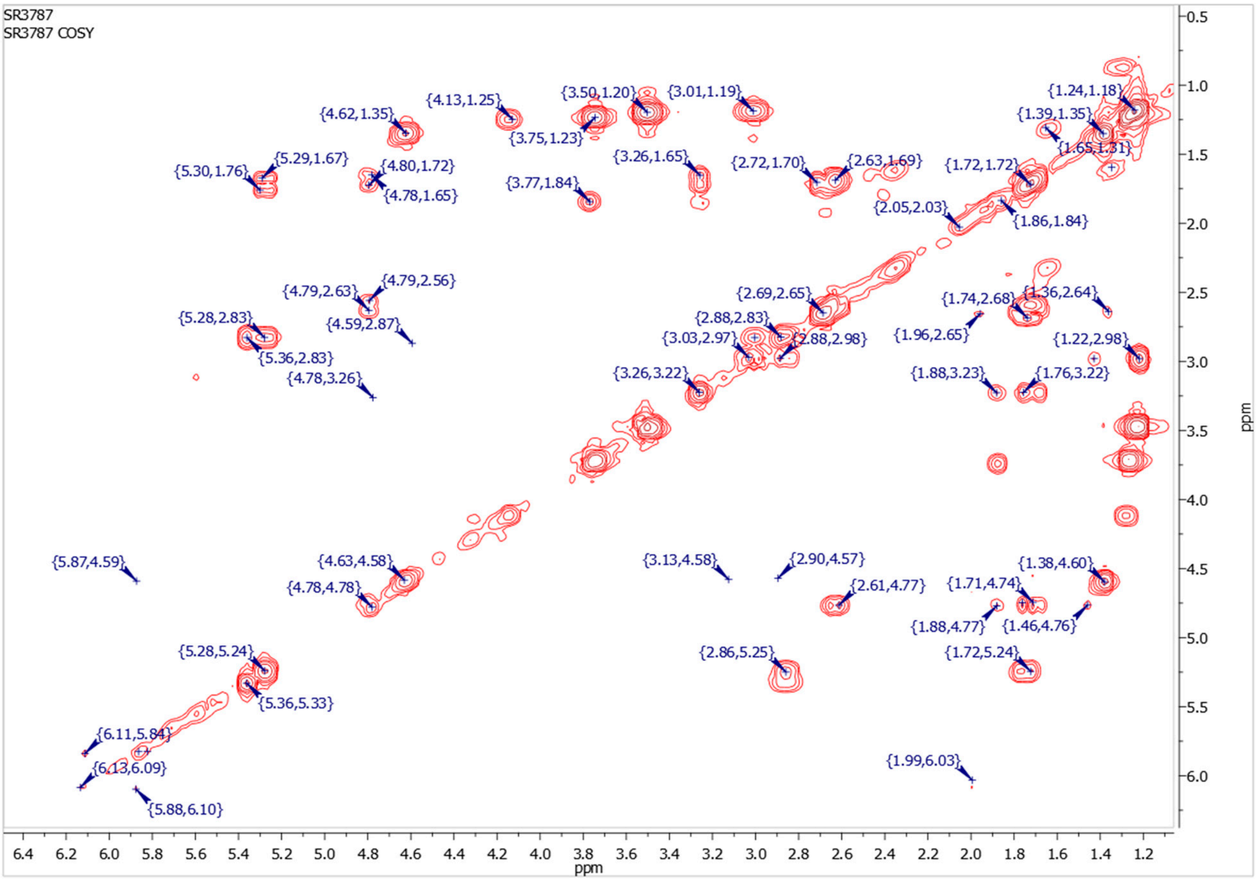

SR3787
SR3787 COSY
{1.24,1.18}
{3.50,1.20}
{3.01,1.19}
{4.13,1.25}
{4.62,1.35}
{1.39,1.35}
{3.75,1.23}
{1.65,1.31}
{5.29,1.67}
{5.30,1.76}
{4.80,1.72}
{4.78,1.65}
{3.26,1.65}
{2.72,1.70}
{2.63,1.69}
{1.72,1.72}
{3.77,1.84}
{2.05,2.03}
{1.86,1.84}
{4.79,2.56}
{4.79,2.63}
{2.69,2.65}
{1.74,2.68}
{1.36,2.64}
{5.28,2.83}
{4.59,2.87}
{2.88,2.83}
{1.96,2.65}
{3.03,2.97}
{2.88,2.98}
{1.22,2.98}
{5.36,2.83}
{4.78,3.26}
{3.26,3.22}
{1.88,3.23}
{1.76,3.22}
{5.87,4.59}
{4.63,4.58}
{3.13,4.58}
{2.90,4.57}
{1.38,4.60}
{4.78,4.78}
{2.61,4.77}
{1.71,4.74}
{1.88,4.77}
{1.46,4.76}
{5.28,5.24}
{2.86,5.25}
{1.72,5.24}
{5.36,5.33}
{6.11,5.84}
{6.13,6.09}
{1.99,6.03}
{5.88,6.10}
ppm
ppm

Panel D

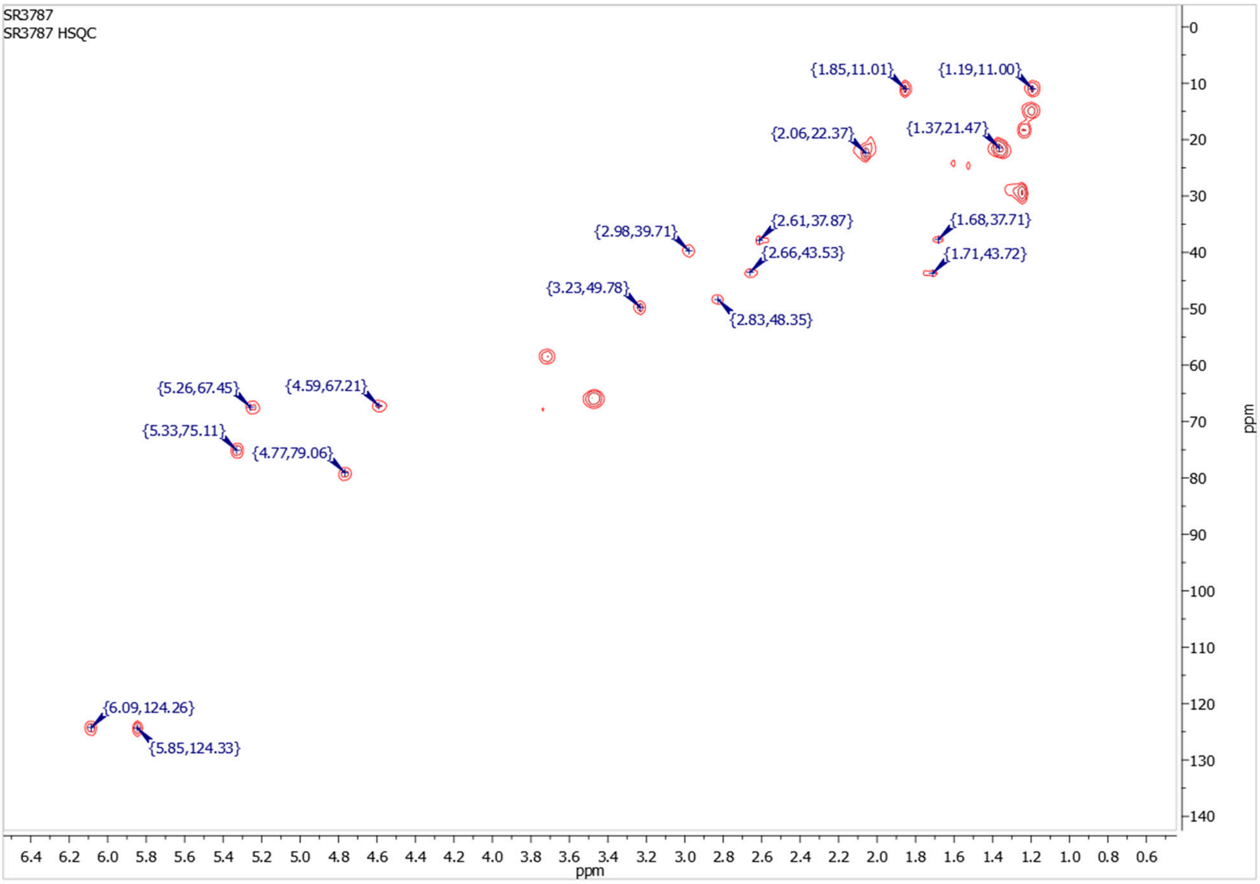
SR3787
SR3787 HSQC
{1.85,11.01}
{1.19,11.00}
{2.06,22.37}
{1.37,21.47}
{2.98,39.71}
{2.61,37.87}
{2.66,43.53}
{1.68,37.71}
{1.71,43.72}
{3.23,49.78}
{2.83,48.35}
{5.26,67.45}
{4.59,67.21}
{5.33,75.11}
{4.77,79.06}
{6.09,124.26}
{5.85,124.33}
ppm
ppm

Panel E

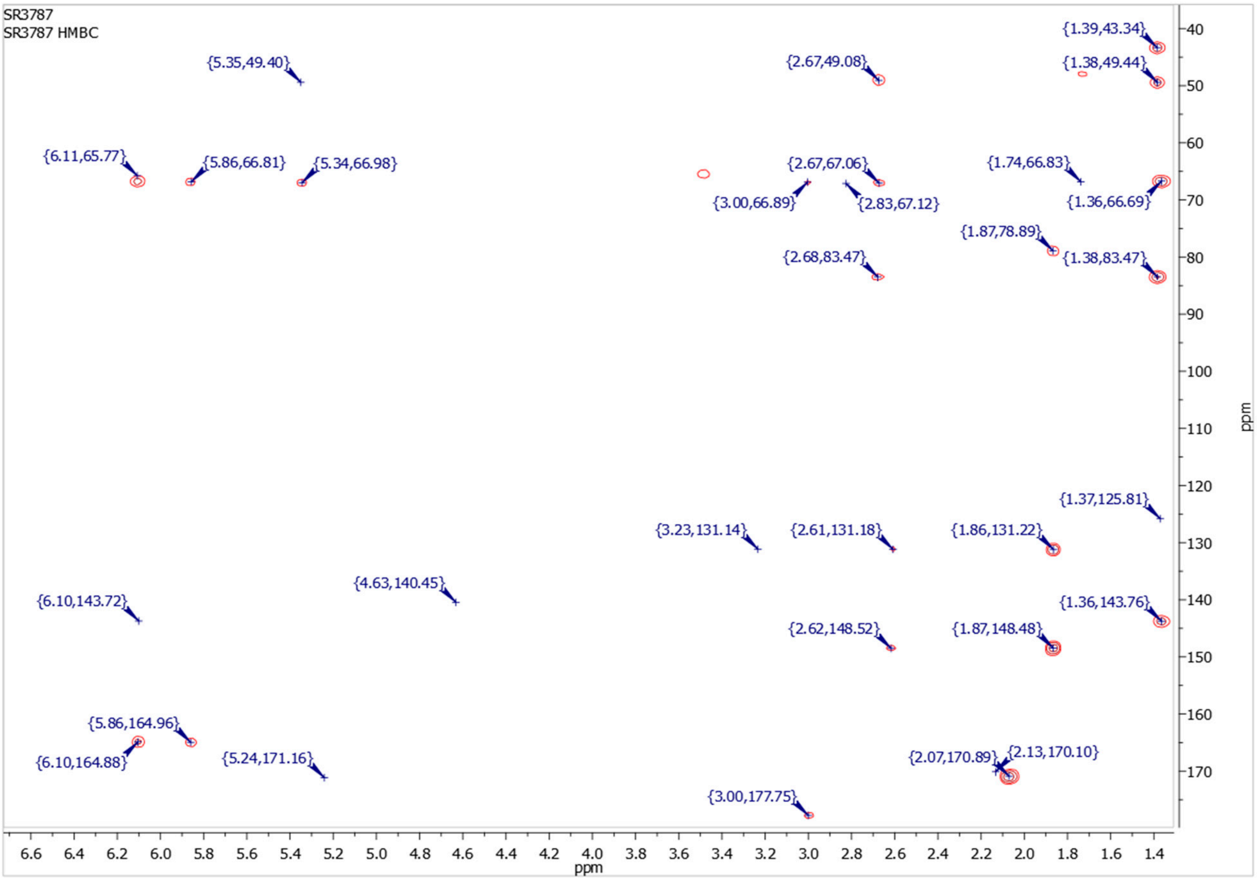
SR3787
SR3787 HMBC
{1.39,43.34}
{5.35,49.40}
{2.67,49.08}
{1.38,49.44}
{6.11,65.77}
{5.86,66.81}
{5.34,66.98}
{2.67,67.06}
{1.74,66.83}
{3.00,66.89}
{2.83,67.12}
{1.36,66.69}
{1.87,78.89}
{2.68,83.47}
{1.38,83.47}
{1.37,125.81}
{3.23,131.14}
{2.61,131.18}
{1.86,131.22}
{4.63,140.45}
{6.10,143.72}
{1.36,143.76}
{2.62,148.52}
{1.87,148.48}
{5.86,164.96}
{6.10,164.88}
{5.24,171.16}
{2.07,170.89}
{2.13,170.10}
{3.00,177.75}
ppm
ppm

Panel F

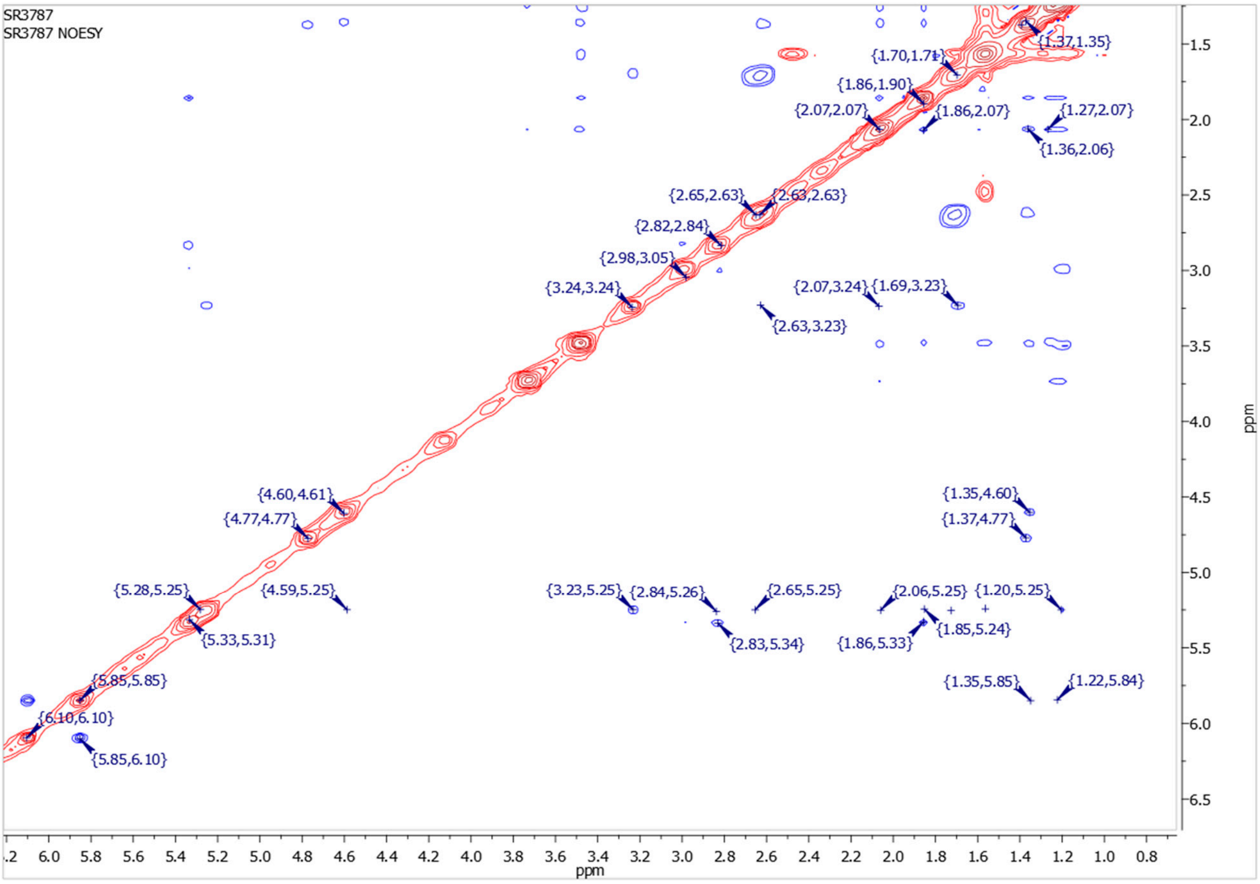

Figure S10. NMR spectra of **SL-3-4** (Panel A→ ^{1}H-NMR, Panel B → ^{13}C-NMR, Panel C → ^{1}H-^{1}H COSY, Panel D →HSQC, Panel E → HMBC, Panel F → NOESY) 400 MHz, $CDCl_3$.

Panel A

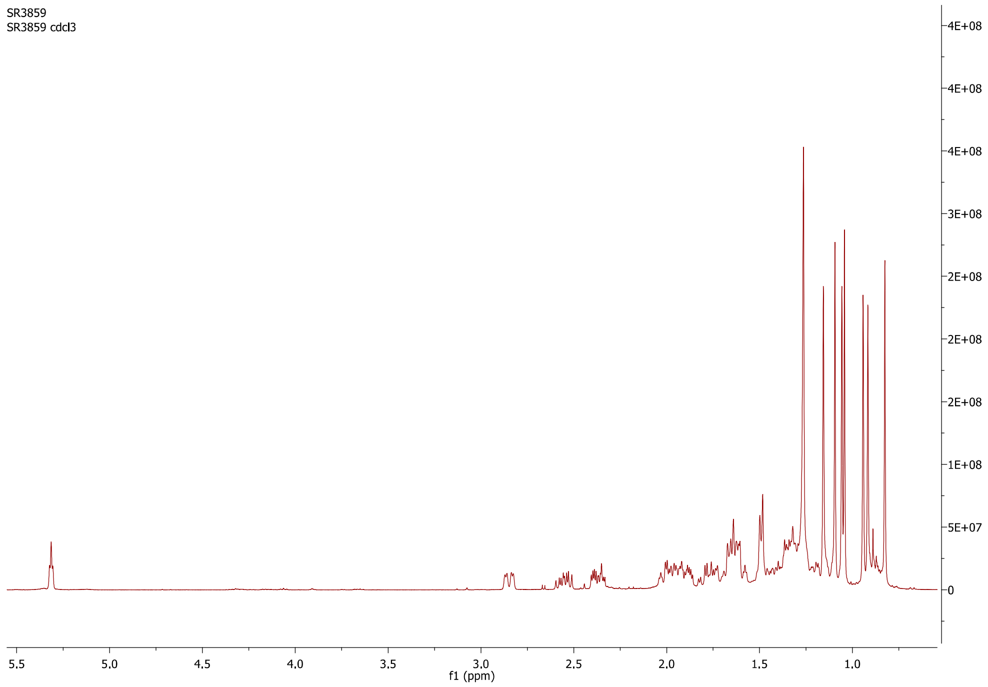

Panel B

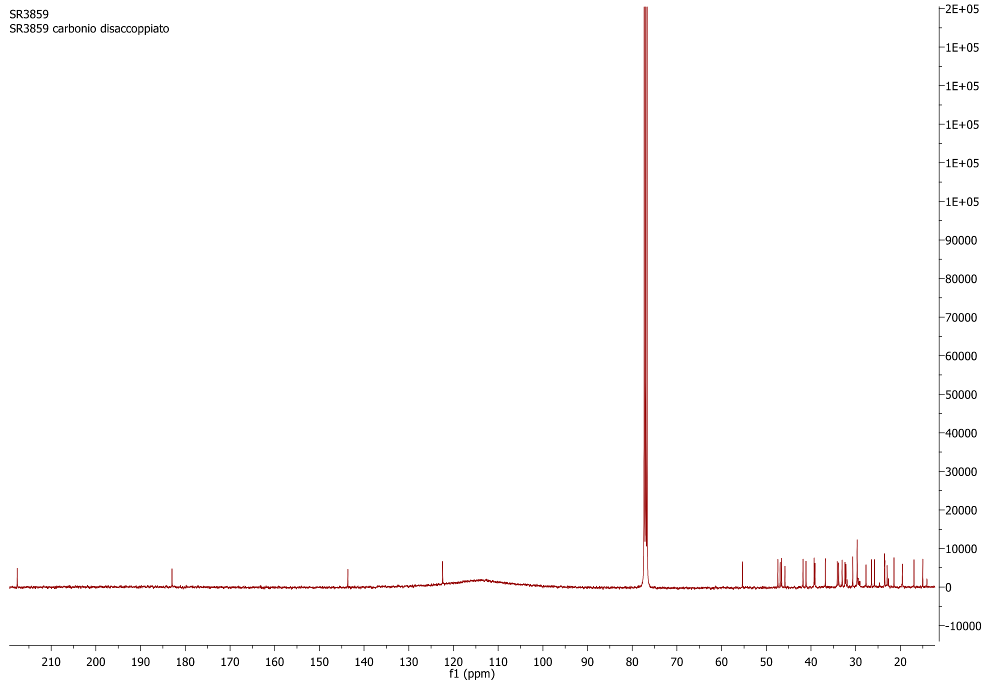

Figure S11. NMR spectra of **5** (Panel A→ ^{1}H-NMR, Panel B → ^{13}C-NMR) 400 MHz, $CDCl_3$.

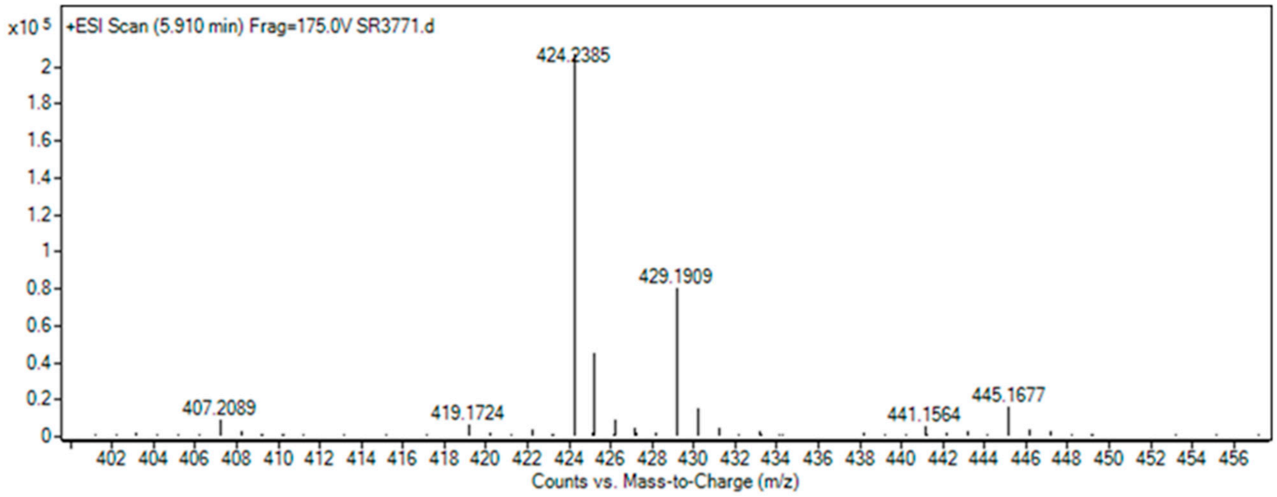

Figure S12. HRESI-MS spectrum of **SL-1** (positive ion mode).

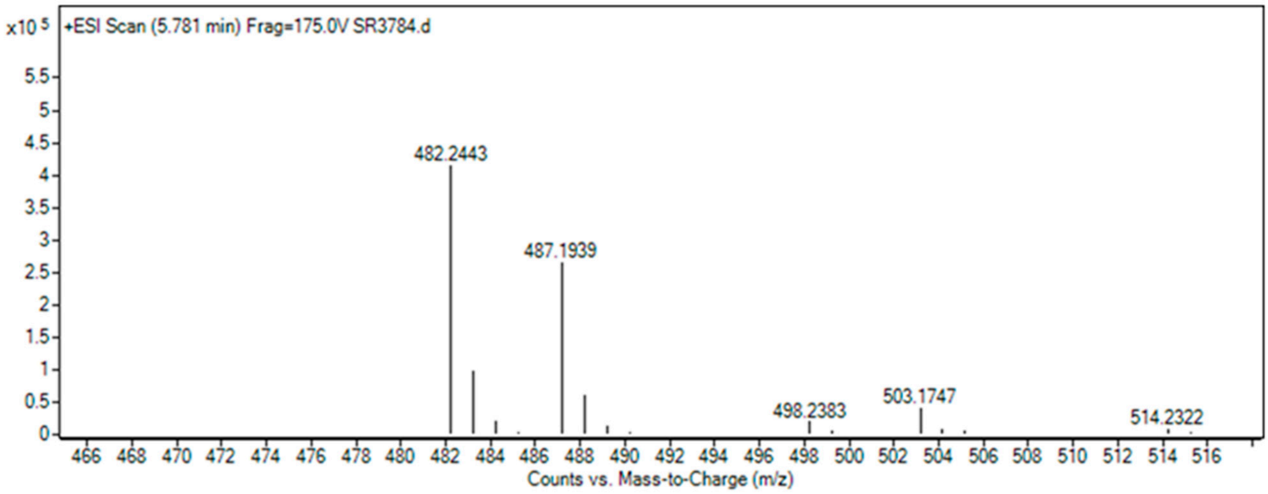

Figure S13. HRESI-MS spectrum of **SL-2** (positive ion mode).

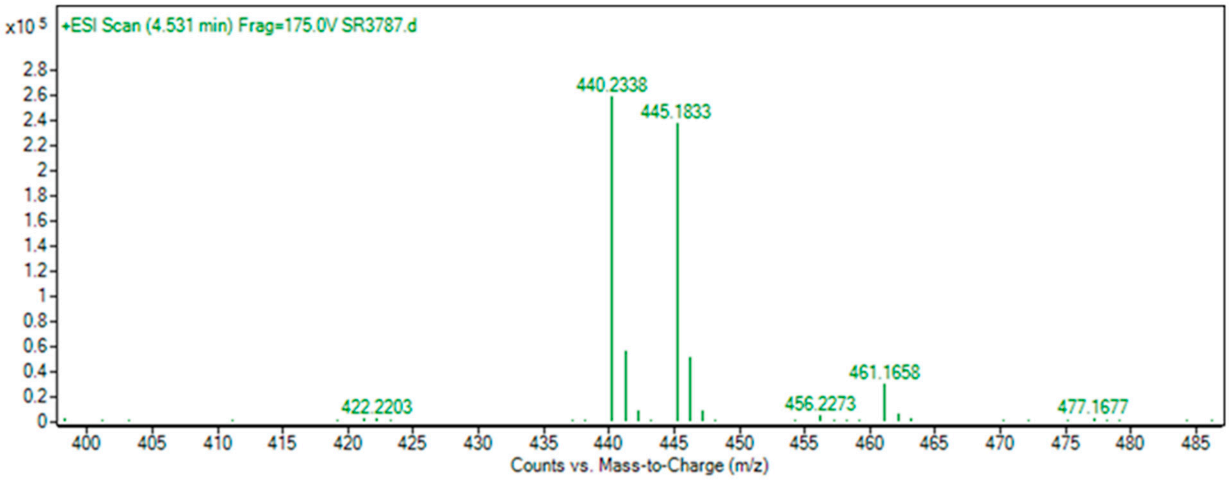

Figure S14. HRESI-MS spectrum of **SL-3** (positive ion mode).

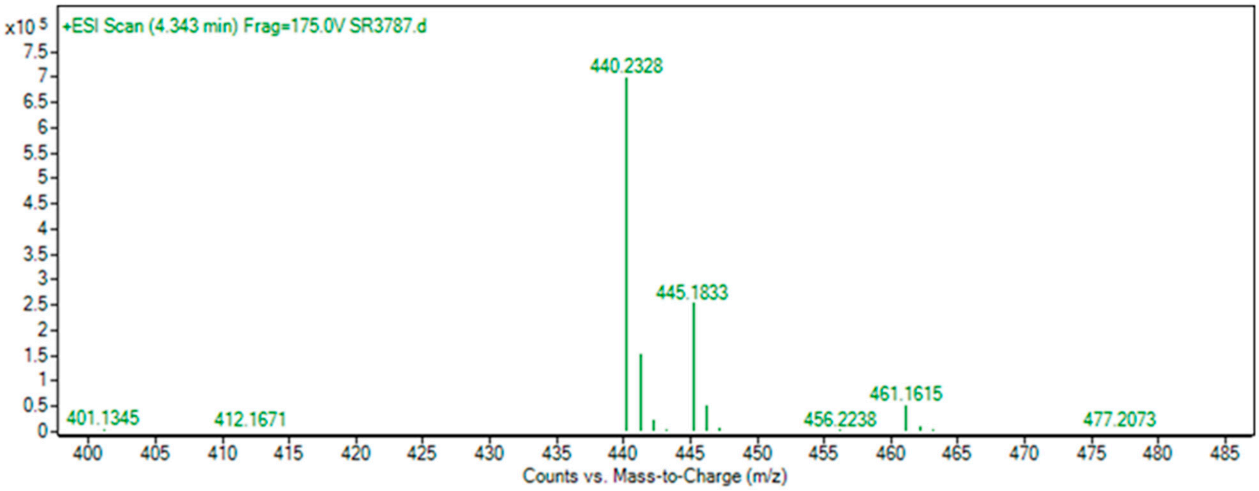

Figure S15. HRESI-MS spectrum of **SL-4** (positive ion mode).
